# Hemochromatosis-Associated Mortality in the United States (1999-2024): A Nationwide Joinpoint Analysis of Trends and Disparities

**DOI:** 10.7759/cureus.111643

**Published:** 2026-06-28

**Authors:** Zenab M Khan, Hamza B Amir, Muhammad Uzair, Astad Y Sidhwa

**Affiliations:** 1 Internal Medicine, Independent Researcher, Karachi, PAK

**Keywords:** age-adjusted mortality rate, cdc wonder, hemochromatosis, hereditary iron overload, icd-10 e83.1, joinpoint regression, mortality trends, racial and ethnic disparities

## Abstract

Introduction: Hemochromatosis is a treatable disorder of iron metabolism in which iron progressively accumulates in the liver, heart, pancreas, and endocrine organs, leading to cirrhosis, hepatocellular carcinoma, cardiomyopathy, diabetes, and other end-organ injury. Prior nationwide U.S. mortality analyses predate the ICD-10 era, and contemporary trend data are limited. We assessed trends and disparities in hemochromatosis-associated mortality in the United States (1999-2024).

Methods: We conducted a retrospective serial cross-sectional analysis of national death-certificate data (STROBE- and RECORD-compliant) using the Centers for Disease Control and Prevention (CDC) Wide-ranging Online Data for Epidemiologic Research (WONDER) Multiple Cause of Death (MCD) database (1999-2020 bridged-race file; 2021-2024 single-race file). Hemochromatosis-associated deaths were identified by ICD-10 code E83.1 (underlying or any contributing cause). Age-adjusted mortality rates (AAMRs) per 100,000 (2000 U.S. standard population) were stratified by sex, race and Hispanic origin, U.S. Census region, age group, state, and urbanization. Temporal trends were assessed with the Joinpoint Regression Program version 6.0.1 (grid search; 0-4 joinpoints; Monte Carlo permutation test, 4,499 permutations; log-linear model), reporting annual percent change (APC) and average annual percent change (AAPC) with 95% confidence intervals (CIs). Segment 1 refers to the period before any identified joinpoint; segment 2 refers to the period after.

Results: Between 1999 and 2024, 16,113 hemochromatosis-associated deaths were recorded; annual counts rose from 562 to 841 (+49.6%). The overall AAMR was 0.21 per 100,000 in 1999, 0.14 in 2015 (nadir), and 0.19 in 2024. Joinpoint regression identified one inflection at 2015 (95% CI, 2011-2018): a 16-year decline (APC -1.75%, p < 0.001) and a nine-year rebound (APC +3.07%, p = 0.002); AAPC -0.04% (p = 0.917) masked this two-segment trajectory. Burden was demographically concentrated: men 10,665 (66.2%), non-Hispanic White (NHW) decedents 13,883 (86.2%), and adults aged ≥65 years 9,706 (61.8% of age-classified deaths) - and the male-to-female AAMR ratio widened from 2.23 to 2.55. The post-joinpoint rebound was confined to NHW decedents (joinpoint 2013; segment-2 APC +3.16%, p < 0.001); non-Hispanic Black and Hispanic rates declined monotonically (AAPCs -1.77% and -2.57%, both p < 0.001). The South and West reproduced the two-segment pattern (joinpoints 2014, 2015); no inflection was detected in the Northeast or Midwest. Crude mortality in the 85-and-older stratum rose from 0.96 to 1.76 per 100,000 (+82.4%), with annual deaths rising from 40 to 113. Chronic liver disease was co-listed on 7,412 (46.0%) certificates, followed by diabetes with 2,408 (14.9%), primary liver cancer 1,739 (10.8%), heart failure 1,715 (10.6%), sepsis 1,193 (7.4%), hepatitis C 745 (4.6%), and cardiomyopathy 674 (4.2%).

Conclusion: This 26-year nationwide analysis identified a statistically significant reversal in U.S. hemochromatosis-associated mortality in 2015, with a 16-year decline followed by a nine-year rebound concentrated in older NHW men. Non-Hispanic Black and Hispanic mortality declined monotonically, widening race and ethnic disparities, and the end-organ comorbidity profile remained stable. Distinguishing coding, cohort, and penetrance contributions will require individual-level data linking HFE genotype, treatment exposure, and clinical outcomes.

## Introduction

Hemochromatosis is a disorder of iron metabolism in which deficient hepcidin activity drives inappropriately elevated intestinal iron absorption and progressive iron deposition in parenchymal tissues of the liver, heart, pancreas, and endocrine organs [1,2]. In contemporary clinical classification, and in the ICD-10 code E83.1 used by U.S. death-certificate data, the term encompasses hereditary HFE-related disease (the predominant form among adults of Northern-European ancestry); non-HFE hereditary forms arising from mutations in HJV, HAMP, TFR2, or SLC40A1; and acquired secondary iron overload from transfusional siderosis, ineffective erythropoiesis, chronic liver disease, and porphyria cutanea tarda [2,3]. Among these, non-HFE hereditary forms are rare (combined population frequency approximately one per five million) [3]; the principal source of interpretive uncertainty in an E83.1-coded mortality analysis is the overlap between HFE-related disease and secondary iron overload attributable to chronic liver disease. Hereditary HFE disease accounts for the large majority of clinically recognized adult cases in this population [3,4]. Feder and colleagues identified HFE on chromosome 6p in 1996 [5], and homozygosity for the HFE p.C282Y missense variant accounts for 80-85% of clinically recognized cases in Northern-European-ancestry populations; compound C282Y/H63D heterozygotes account for most of the remainder [3,4].

Within Northern-European-ancestry populations, C282Y homozygote prevalence approaches one in 220-250, placing hereditary hemochromatosis among the most common inherited disorders in this group [4]. The HFE p.C282Y heterozygote frequency is approximately 9% in European populations and essentially absent in African, Asian, and most Indigenous populations [6]. The Hemochromatosis and Iron Overload Screening (HEIRS) Study, which screened nearly 100,000 U.S. primary-care adults, documented a marked ancestry gradient in C282Y homozygote prevalence: 4.4 per 1,000 non-Hispanic Whites, 0.27 per 1,000 Hispanics, and 0.14 per 1,000 African Americans, a 16-fold gradient between non-Hispanic Whites and Hispanics and a 31-fold gradient between non-Hispanic Whites and African Americans [7].

Clinical sequelae of untreated iron overload include hepatic fibrosis, cirrhosis, hepatocellular carcinoma, cardiomyopathy, diabetes mellitus, hypogonadism, and arthropathy; in established cirrhosis, the relative risk of hepatocellular carcinoma is approximately 20, with an annual incidence of 3-4% [3,4]. Penetrance is incomplete and markedly sex-dependent because menstrual and gestational iron losses attenuate iron accumulation in premenopausal women. Direct U.S. penetrance estimates are scarce, and the best current quantification comes from the UK Biobank cohort - the largest HFE-genotyped longitudinal dataset available, although its generalizability to U.S. populations is partial given differences in ancestry composition, healthcare access, and cohort recruitment. In the UK Biobank, Pilling and colleagues reported that, among 2,890 p.C282Y homozygotes (1,294 men and 1,596 women), hemochromatosis was diagnosed during follow-up in 21.7% of men versus 9.8% of women [8]. Subsequent analyses in the same cohort demonstrated excess all-cause mortality in homozygous men (hazard ratio 1.29; 95% CI, 1.12-1.48) [9] and a hazard ratio of 10.5 (95% CI, 6.6-16.7) for primary hepatic malignancy in this group [10], with no statistically significant homozygosity-associated risks in women [9,10]. Importantly, hereditary hemochromatosis is treatable: therapeutic phlebotomy, instituted before cirrhosis, can restore normal life expectancy [11] and remains first-line management in guidelines from the American Association for the Study of Liver Diseases (AASLD) and the American College of Gastroenterology (ACG) [3,4].

Despite a defined pathophysiology, identifiable end-organ sequelae, and effective treatment, contemporary nationwide surveillance of hemochromatosis-associated mortality in the United States is limited. The foundational population analysis, Yang and colleagues' examination of Multiple-Cause Mortality Data for 1979-1992, reported substantially elevated proportionate mortality ratios for liver neoplasms, chronic liver disease, and cardiomyopathy among hemochromatosis decedents relative to non-hemochromatosis decedents [12]. Brown and colleagues subsequently analyzed National Hospital Discharge Survey hospitalization data for 1979-1997 [13]. Both predate widespread clinical HFE genotyping, noninvasive MRI quantification of hepatic iron, and the U.S. transition from ICD-9 to ICD-10 mortality coding in 1999; NCHS comparability ratios for ICD-9 275.0 to ICD-10 E83.1 are not routinely published for lower-frequency causes, which prevents direct bridging across these literatures. More recently, Abou Yassine and colleagues reported a 79% rise in hemochromatosis-related hospitalizations in the Nationwide Inpatient Sample between 2002 and 2010 (from 34.5 to 61.4 per 100,000 hospitalizations, as reported by the authors), attributed in part to increased clinical recognition [14]; whether this inpatient increase has been accompanied by parallel or divergent mortality changes has not been established.

Two analytic considerations compound this surveillance gap. First, standard age-adjusted-rate reporting cannot detect mid-period trend changes. Recent joinpoint analyses of Centers for Disease Control and Prevention (CDC) Wide-ranging Online Data for Epidemiologic Research (WONDER) data have documented two-segment trajectories across 1999-2024 for several chronic conditions, including sudden cardiac death [15], hemolytic anemia [16], and amyloidosis co-coded with cardiovascular disease [17], among others, although not all such analyses yield inflections, and the method itself does not predetermine what will be found. Whether hemochromatosis-associated mortality exhibits a comparable inflection and whether any inflection is shared across demographic strata is unknown. Second, hemochromatosis is often recorded as a contributing rather than underlying cause of death, because decedents frequently die of end-organ sequelae listed as the underlying cause. Analyses restricted to the underlying-cause file therefore materially underestimate burden, and joint analysis of underlying and contributing causes has been argued to provide more complete ascertainment [18]. Together, these gaps leave three substantive questions unanswered for the post-1999 period: whether overall hemochromatosis-associated mortality has risen, fallen, or shifted direction; whether any change has been shared across demographic strata defined by sex, race and Hispanic origin, region, urbanization, and age; and whether the classical end-organ comorbidity profile documented by Yang and colleagues has persisted as clinical recognition has expanded.

We therefore conducted a descriptive, hypothesis-generating analysis of hemochromatosis-associated mortality in the United States across 1999-2024, using CDC WONDER Multiple Cause of Death (MCD) data and joinpoint regression [19]. The primary question was whether nationwide trend inflections have occurred in the age-adjusted mortality rate, and if so, whether such inflections are shared across demographic strata. Secondary aims were to quantify disparities by sex, race and Hispanic origin, U.S. Census region, state, urbanization, and age group; to describe the comorbidity profile of hemochromatosis-associated deaths across chronic liver disease, liver cancer, heart failure, cardiomyopathy, sepsis, diabetes, and hepatitis C; and to place the observed trends alongside contemporaneous CDC WONDER-based analyses of related chronic conditions. Implications for current screening recommendations are addressed in the Discussion. To our knowledge, this is the first nationwide joinpoint analysis of hemochromatosis-associated mortality spanning the full ICD-10 era.

## Materials and methods

Study design and reporting

We conducted a retrospective, serial cross-sectional analysis of national death-certificate data to characterize trends in hemochromatosis-associated mortality among United States residents from 1999 through 2024. Reporting follows the Strengthening the Reporting of Observational Studies in Epidemiology (STROBE) statement [20] and the REporting of studies Conducted using Observational Routinely-collected Data (RECORD) extension for studies using routinely collected health data [21].

Data source

Mortality data were obtained from the CDC WONDER MCD database, which compiles records from the National Vital Statistics System maintained by the National Center for Health Statistics [22]. Two MCD files were queried to span the full study period: the 1999-2020 bridged-race file and the 2018-2024 single-race file. Data for 2021 through 2024 were obtained from the latter; overlapping years in the single-race file were not used, ensuring no record was double-counted. Race and Hispanic-ethnicity categories were harmonized across the bridged-race and single-race coding schemes by restricting analyses to population groups whose definitions are directly comparable between the two schemes.

Case definition

Hemochromatosis-associated deaths were identified from records with International Classification of Diseases, Tenth Revision (ICD-10) code E83.1 (Disorders of iron metabolism) listed anywhere on the death certificate, as either the underlying or any contributing cause of death. Multiple-cause ascertainment was prespecified because hemochromatosis frequently appears on death certificates as a contributing diagnosis alongside end-organ manifestations such as hepatic cirrhosis, hepatocellular carcinoma, cardiomyopathy, and diabetes mellitus; analyses restricted to the underlying cause substantially underestimate disease burden [12,18]. U.S. death certificates are coded using ICD-10 (international), not the ICD-10-CM clinical modification used for hospital encounter coding; CDC WONDER returns mortality data at the 3-character ICD-10 level for E83.1, which encompasses all disorders of iron metabolism without subdivision into hemochromatosis-specific (ICD-10-CM E83.11) versus other (E83.10, E83.19) subcategories.

Population and stratification

All U.S. residents with a qualifying death record between January 1, 1999, and December 31, 2024, were included. Mortality was examined overall and stratified by sex (male, female), race and Hispanic ethnicity (non-Hispanic White, non-Hispanic Black or African American, Hispanic or Latino), and U.S. Census region (Northeast, Midwest, South, West). Non-Hispanic Asian/Pacific Islander and non-Hispanic American Indian/Alaska Native subgroups had fewer than 10 deaths in most annual cells and were not analyzed as separate strata. For descriptive age-specific analyses, decedents were grouped into 10-year strata as provided by CDC WONDER.

Statistical analysis

Age-adjusted mortality rates (AAMRs) per 100,000 population were obtained directly from CDC WONDER, computed by the direct method with the 2000 U.S. standard population as the reference [23]. Crude rates were computed as total deaths divided by the sum of annual mid-year U.S. resident population estimates over the period of interest, expressed per 100,000 person-years. Per NCHS convention, rates based on fewer than 20 deaths are flagged as statistically unreliable (relative standard error ≥ 23%), and counts representing fewer than 10 deaths are suppressed [22]. Years flagged unreliable were excluded from the Joinpoint input for the affected subgroup; Hispanic AAMRs for 1999, 2005, and 2015 were excluded on this basis, yielding a 23-year analytic series spanning 2000-2024 with 2005 and 2015 excluded.

Temporal trends in AAMRs were evaluated using the National Cancer Institute Joinpoint Regression Program, version 6.0.1 (Statistical Research and Applications Branch, National Cancer Institute). Each subgroup series was modeled on the natural-log scale with an uncorrelated error structure, using the standard errors provided by the CDC WONDER. Models were fit by Grid Search with a minimum and maximum of zero and four joinpoints, a minimum of two observations from any joinpoint to the series endpoints, and a minimum of two observations between adjacent joinpoints. The optimal number of joinpoints was selected by the Monte Carlo permutation test of Kim and colleagues with 4,499 permutations and an overall significance level of 0.05 [19].

For each segment, the annual percent change (APC) was computed from the segment slope. The 1999-2024 average annual percent change (AAPC) for each subgroup was computed as a geometric weighted average of segment-specific APCs, with weights proportional to segment length in years. Parametric 95% CIs were used throughout, applied to joinpoint years, segment APCs, and AAPCs as implemented in Joinpoint 6.0.1; these are asymptotically valid given the ≥ 23-year observation series in every subgroup. An APC or AAPC was considered statistically significant when its 95% CI excluded zero (two-sided α = 0.05); in tables and figures, asterisks denote values meeting this criterion. When a joinpoint divides a series into two distinct portions, we refer to the period before the joinpoint as segment 1 and the period after the joinpoint as segment 2; these segments are described by their own segment-specific APCs. Ten Joinpoint analyses were performed with identical specifications - overall, by sex (two strata), by race and Hispanic origin (three strata), and by Census region (four strata) - to permit cross-stratum comparison.

Because non-significant AAPCs can mask clinically meaningful trend reversals within the study period, segment-specific APCs were interpreted as the primary trend measure in subgroups where the permutation test identified one or more joinpoints; AAPCs are reported for completeness and for cross-subgroup comparison.

Because 10 Joinpoint analyses were performed at a per-test significance level of 0.05 with no formal multiple-comparison correction applied, we additionally report the impact of Bonferroni adjustment (corrected α = 0.005). Under this adjustment, the principal findings of the overall analysis (segment-1 APC, p < 0.001; segment-2 APC, p = 0.002), the male trajectory (segment-1, p < 0.001; segment-2, p < 0.001), the non-Hispanic White trajectory (segment-1, p = 0.003; segment-2, p < 0.001), the non-Hispanic Black AAPC (p < 0.001), the Hispanic AAPC (p < 0.001), and the South and West segment-1 APCs (both p ≤ 0.004) would all remain significant. Borderline subgroup findings - female segment-1 (p = 0.009), female segment-2 (p = 0.049), South segment-2 (p = 0.013), and West segment-2 (p = 0.011) - would not survive Bonferroni correction and should be interpreted with the family-wise error rate in mind.

Secondary descriptive analyses

The co-occurrence of E83.1 with selected comorbid conditions on the same death certificate was tabulated to characterize the clinical context of hemochromatosis-associated mortality. The conditions examined were chronic liver disease (K70-K76), primary liver cancer (C22), hepatitis C infection (B17.1, B18.2), cardiomyopathy (I42), heart failure (I50), diabetes mellitus (E10-E14), sepsis (A40-A41), and gout (M10). For conditions with most year-level cells suppressed (e.g., gout, M10), the unsuppressed 26-year period total was obtained from the CDC WONDER aggregate row, which sums all qualifying deaths across the requested dimensions regardless of within-cell suppression at finer stratifications. Counts are reported as absolute numbers and as proportions of total E83.1-associated deaths and are descriptive only; no inferential trend analysis was performed for co-occurring conditions.

Software

All mortality data were extracted from CDC WONDER (https://wonder.cdc.gov/mcd.html) [22]. Joinpoint regression was fit using the Joinpoint Regression Program, version 6.0.1 (Statistical Research and Applications Branch, National Cancer Institute, Bethesda, MD). Trend figures were generated in Python (version 3.x) from Joinpoint Program output files using segment-specific fitted values of the form exp(β₀ + β₁·year), where β₀ and β₁ are the segment-specific intercept and slope from the Joinpoint Model Estimates export. Descriptive tabulations and figure production used Python (pandas, numpy, and matplotlib).

Ethics

Because the data are de-identified, publicly available, and reported in aggregate by CDC WONDER, this analysis did not constitute human-subjects research, and institutional review board approval was not required.

## Results

Overall mortality burden and descriptive distribution

Between January 1, 1999, and December 31, 2024, 16,113 hemochromatosis-associated deaths (ICD-10 E83.1, listed as any cause of death) were recorded in United States residents. Of these, 12,850 (79.7%) occurred during 1999-2020 (bridged-race file) and 3,263 (20.3%) during 2021-2024 (single-race file), corresponding to an annual mean of 584 deaths during 1999-2020 and 816 deaths during 2021-2024. Annual counts ranged from a low of 523 in 2007 to a high of 841 in 2024 (Appendix D). Compared with the 562 deaths recorded in 1999, the 841 deaths in 2024 represented an absolute increase of 279 deaths (49.6%) in the annual crude count, despite a net-flat age-adjusted rate across the full period.

The demographic, geographic, and urbanization distribution of decedents is summarized in Table 1. Sex was reported for 15,850 deaths (98.4%); cells with fewer than 10 deaths in the 1999-2020 year × sex × race export were suppressed, accounting for the 263-death shortfall. Among the sex-reported deaths, 10,665 (67.3%; 66.2% of all 16,113) occurred among males and 5,185 (32.7%; 32.2% of all) among females. Race and Hispanic origin were comparable across the bridged-race and single-race files for 15,630 deaths (97.0%): non-Hispanic White decedents accounted for 13,883 (86.2%), non-Hispanic Black for 1,086 (6.7%), and Hispanic or Latino for 661 (4.1%). Age at death was assigned for 15,703 deaths (97.5%), with 8,046 (51.2% of age-classified; 49.9% of all 16,113) occurring in those aged 65-84 years, 1,660 (10.6% of age-classified) in those aged 85 or older, and 1,046 (6.7% of age-classified) before age 45. By the U.S. Census region, the South contributed the largest absolute burden (5,459 deaths; 33.9%), followed by the West (4,167; 25.9%), the Midwest (3,523; 21.9%), and the Northeast (2,964; 18.4%); region totals summed to 16,113 exactly. By the 2013 NCHS Urban-Rural Classification, 12,822 deaths (79.6%) occurred in metropolitan counties and 3,291 (20.4%) in non-metropolitan counties, with medium-metro the largest single category (3,862; 24.0%). Annual death counts for every analyzed stratum across 1999-2024 are shown in Appendix D.

**Table 1:** Demographic, geographic, and urbanization distribution of hemochromatosis-related deaths (ICD-10 E83.1) in the United States, 1999-2024 Deaths are counts of records with ICD-10 code E83.1 listed as any cause of death on U.S. death certificates, accessed via CDC WONDER Multiple Cause of Death files (Bridged-Race file 1999-2020; Single-Race file 2021-2024). Percentages are of the overall 16,113 hemochromatosis-related deaths. Stratum counts may sum to less than 16,113 owing to CDC confidentiality suppression of cells with fewer than 10 deaths. Crude rate per 100,000 population computed as total deaths divided by the total population-years times 100,000; 95% CI by normal approximation. AAMR values are observed age-adjusted mortality rates for the first and last years of each stratum's joinpoint analysis, standardized to the 2000 U.S. standard population. Sex counts (male + female) sum to 15,850; the 263-death shortfall reflects suppression in the 1999-2020 year × sex × race export. Race/Hispanic origin counts sum to 15,630 (97.0% of total); the remaining 3.0% occurred in groups not analyzed as separate strata. For Hispanic or Latino, the joinpoint analysis used 23 observation years (2000-2024, excluding 2005 and 2015); the AAMR shown for "1999" corresponds to 2000. Age-group totals sum to 15,703 (97.5%); the 410-death shortfall reflects suppression. The CDC WONDER 2021-2024 Single-Race export reports "not available" for urbanization-stratified populations in 2022-2024. Abbreviations: AAMR, age-adjusted mortality rate; CI, confidence interval; ICD-10, International Classification of Diseases, 10th Revision; NCHS, National Center for Health Statistics

Characteristic	Deaths, N (%)	Population-years	Crude rate per 100,000 (95% CI)	AAMR 1999 (per 100,000)	AAMR 2024 (per 100,000)
Overall					
Overall (U.S., all ages)	16,113 (100.0)	8,086,563,832	0.20 (0.20-0.20)	0.21	0.19
Sex					
Male	10,665 (66.2)	3,813,317,246	0.28 (0.27-0.28)	0.29	0.28
Female	5,185 (32.2)	3,906,816,212	0.13 (0.13-0.14)	0.13	0.11
Race and Hispanic origin					
Non-Hispanic White	13,883 (86.2)	5,178,106,463	0.27 (0.26-0.27)	0.22	0.24
Non-Hispanic Black	1,086 (6.7)	1,033,125,500	0.11 (0.10-0.11)	0.14	0.08
Hispanic or Latino	661 (4.1)	1,286,778,199	0.05 (0.05-0.06)	0.10	0.06
Age group (10-year bands)					
15-24 years	30 (0.2)	210,606,973	0.01 (0.01-0.02)	0.03	—
25-34 years	300 (1.9)	943,651,017	0.03 (0.03-0.04)	0.04	0.03
35-44 years	716 (4.4)	1,108,316,424	0.06 (0.06-0.07)	0.09	0.05
45-54 years	1,725 (10.7)	1,089,970,438	0.16 (0.15-0.17)	0.17	0.13
55-64 years	3,226 (20.0)	934,829,484	0.35 (0.33-0.36)	0.37	0.40
65-74 years	4,176 (25.9)	648,043,148	0.64 (0.62-0.66)	0.88	0.69
75-84 years	3,870 (24.0)	369,898,963	1.05 (1.01-1.08)	1.07	1.13
85+ years	1,660 (10.3)	144,605,638	1.15 (1.09-1.20)	0.96	1.76
U.S. Census region					
Northeast	2,964 (18.4)	1,442,011,618	0.21 (0.20-0.21)	0.21	0.19
Midwest	3,523 (21.9)	1,742,256,120	0.20 (0.20-0.21)	0.18	0.18
South	5,459 (33.9)	3,016,550,585	0.18 (0.18-0.19)	0.18	0.18
West	4,167 (25.9)	1,885,745,509	0.22 (0.21-0.23)	0.25	0.20
2013 NCHS urbanization classification					
Large Central Metro	3,463 (21.5)	2,163,927,630	0.16 (0.15-0.17)	0.16	NA
Large Fringe Metro	3,619 (22.5)	1,740,655,115	0.21 (0.20-0.21)	0.21	NA
Medium Metro	3,862 (24.0)	1,470,489,738	0.26 (0.25-0.27)	0.21	NA
Small Metro	1,878 (11.7)	650,214,322	0.29 (0.28-0.30)	0.21	NA
Micropolitan (non-metro)	1,929 (12.0)	617,584,069	0.31 (0.30-0.33)	0.24	NA
NonCore (non-metro)	1,362 (8.5)	435,370,172	0.31 (0.30-0.33)	0.22	NA

Overall temporal trend in age-adjusted mortality

**Figure 1:**
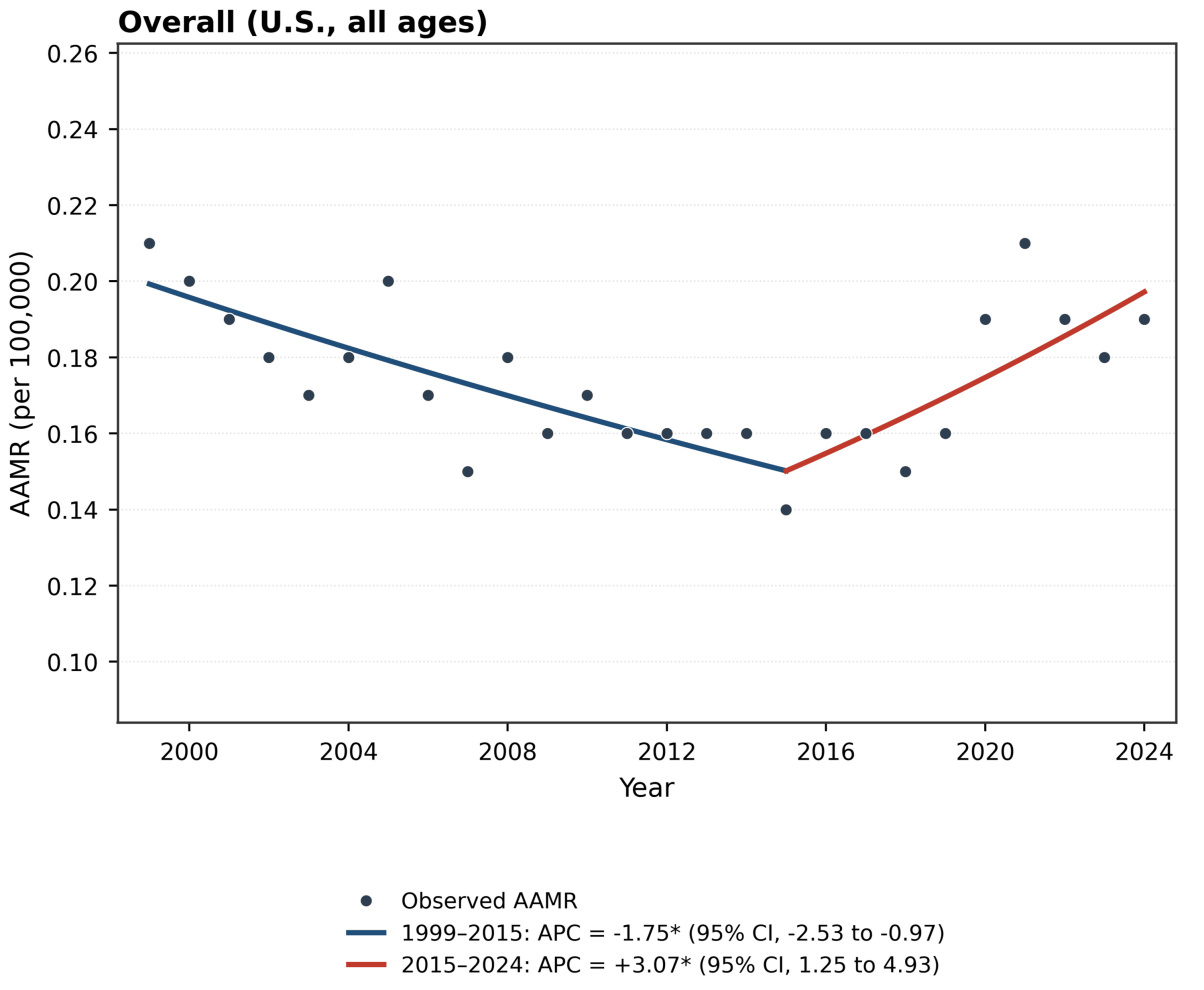
Age-adjusted mortality rates for hemochromatosis-related deaths (ICD-10 E83.1), United States, 1999-2024 Filled circles show observed annual age-adjusted mortality rates (AAMRs, per 100,000, 2000 U.S. standard population). Solid segments show fitted joinpoint regression (NCI Joinpoint version 6.0.1, log-linear model; Monte Carlo permutation test, 4,499 permutations; α = 0.05). One joinpoint was identified in 2015 (95% CI, 2011-2018). Segment 1 (1999-2015): APC = -1.75% (95% CI, -2.53 to -0.97), p < 0.001. Segment 2 (2015-2024): APC = +3.07% (95% CI, +1.25 to +4.93), p = 0.002. Average annual percent change over 1999-2024: -0.04% (95% CI, -0.81 to +0.73), p = 0.917. *APC significantly different from zero at α = 0.05. Abbreviations: AAMR, age-adjusted mortality rate; CI, confidence interval; NCI: National Cancer Institute

The overall age-adjusted mortality rate (AAMR) was 0.21 per 100,000 in 1999, declined to a nadir of 0.14 per 100,000 in 2015, and recovered to 0.19 per 100,000 by 2024 (Figure 1; observed and joinpoint-fitted annual values are tabulated in Appendix A). Joinpoint regression identified a single joinpoint at 2015 (95% CI, 2011-2018), partitioning the 26-year series into two statistically distinct segments (Table 2). Full Joinpoint model-selection statistics, including segment-specific intercepts and slopes for every stratum, are shown in Appendix B; the corresponding Monte Carlo permutation test sequence used to determine the optimal number of joinpoints in each stratum is shown in Appendix C. The AAMR declined at an annual percent change (APC) of -1.75% (95% CI, -2.53 to -0.97; p < 0.001) from 1999 to 2015 - a cumulative observed decline of 33.3% - and then rose at +3.07% per year (95% CI, +1.25 to +4.93; p = 0.002) from 2015 to 2024, a cumulative observed increase of 35.7% from the nadir. The average annual percent change (AAPC) across the full 1999-2024 period was -0.04% (95% CI, -0.81 to +0.73; p = 0.917), not statistically different from zero; this net-flat AAPC reflects the offset between the declining and rising segments rather than a stable underlying rate.

Trends by sex

**Figure 2:**
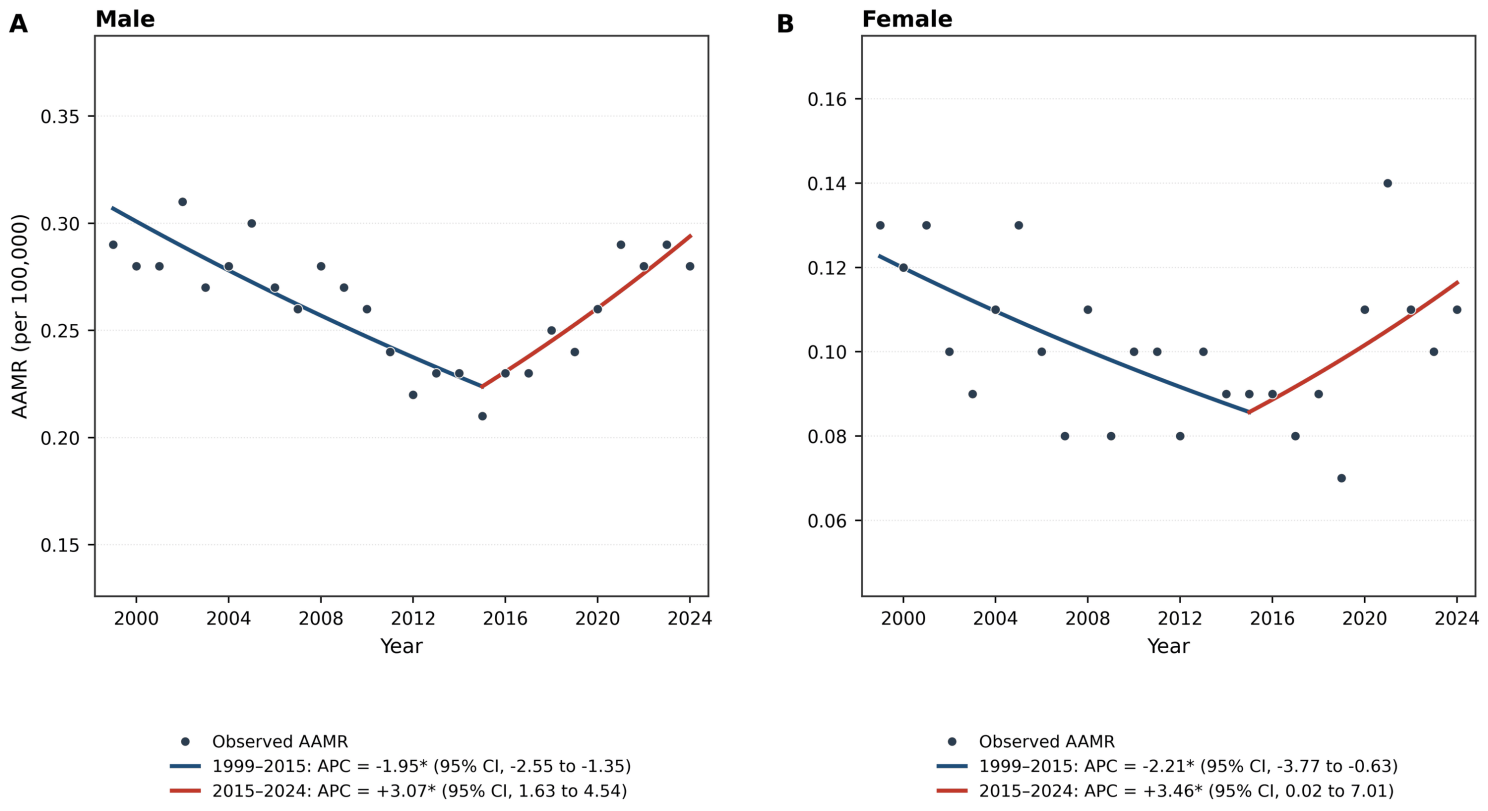
Age-adjusted mortality rates for hemochromatosis-related deaths by sex, United States, 1999–2024. (A) Male. (B) Female. Both sexes showed a significant declining APC through 2015 (male APC₁ = -1.95%, 95% CI, -2.55 to -1.35; female APC₁ = -2.21%, 95% CI, -3.77 to -0.63), followed by a significant rebound 2015-2024 (male APC₂ = +3.07%, 95% CI, +1.63 to +4.54; female APC₂ = +3.46%, 95% CI, +0.02 to +7.01). Joinpoint years: male 2015 (95% CI, 2013-2018); female 2015 (95% CI, 2006-2020). AAPCs for 1999-2024 were not statistically significant (male: -0.17%, p = 0.576; female: -0.21%, p = 0.785). *APC significantly different from zero at α = 0.05 (95% CI excludes zero). Abbreviations: APC, annual percent change; CI, confidence interval; AAPC, average annual percent change

Male AAMR was 0.29 per 100,000 in 1999 and 0.28 per 100,000 in 2024; female AAMR was 0.13 per 100,000 in 1999 and 0.11 per 100,000 in 2024 (Figure 2; Table 2; annual fitted values, Appendix A). The male-to-female AAMR ratio was 2.23 in 1999 and 2.55 in 2024. For males, a single joinpoint was identified at 2015 (95% CI, 2013-2018): AAMR declined at -1.95% per year (95% CI, -2.55 to -1.35; p < 0.001) from 1999 to 2015 and then rose at +3.07% per year (95% CI, +1.63 to +4.54; p < 0.001) from 2015 to 2024, with an AAPC of -0.17% (95% CI, -0.77 to +0.43; p = 0.576). For females, a single joinpoint was identified at 2015 (95% CI, 2006-2020): AAMR declined at -2.21% per year (95% CI, -3.77 to -0.63; p = 0.009) from 1999 to 2015 and then rose at +3.46% per year (95% CI, +0.02 to +7.01; p = 0.049) from 2015 to 2024. The second female segment reached nominal statistical significance only, with the lower confidence bound abutting zero and a wide interval indicating substantial uncertainty about magnitude. The female AAPC was -0.21% (95% CI, -1.70 to +1.30; p = 0.785). Both sexes followed the same qualitative two-segment pattern of significant decline through 2015, followed by a rise, with comparable proportional magnitudes in each segment (segment-1 APC -1.95% to -2.21%; segment-2 APC +3.07% to +3.46%). Permutation-test details for both sex strata are reported in Appendix C.

**Table 2:** Joinpoint regression analysis of age-adjusted mortality rates for hemochromatosis-related deaths (ICD-10 E83.1) in the United States, 1999–2024, by sex, race and Hispanic origin, and U.S. Census region Age-adjusted mortality rates per 100,000, standardized to the 2000 U.S. standard population using the direct method. For Male and Female, counts are aggregated from the Year×Sex×Race (1999–2020) and Year×Sex (2021–2024) CDC WONDER exports; sex counts sum to 15,850 because cells with <10 deaths were suppressed in the 1999–2020 bridged-race file. For Hispanic or Latino, the joinpoint analysis used 2000–2024 (23 observation years; years 1999, 2005, and 2015 were flagged by CDC WONDER as unreliable [<20 deaths] and excluded); the "AAMR, 1999" column reports the 2000 rate. Joinpoint regression fit using the NCI Joinpoint Regression Program, version 6.0.1, with a log-linear model, uncorrelated errors, grid-search, and Monte Carlo permutation test for model selection (4,499 permutations; overall significance level α = 0.05). The minimum number of joinpoints allowed was zero and the maximum was four; at least two observations were required between joinpoints and between a joinpoint and the endpoints. Parametric 95% confidence intervals were used for APC and AAPC. *APC or AAPC significantly different from zero at α = 0.05 (two-sided). Abbreviations: AAMR, age-adjusted mortality rate; APC, annual percent change; AAPC, average annual percent change; CI, confidence interval

Stratum	Deaths, N (%)	AAMR 1999 (per 100,000)	AAMR 2024 (per 100,000)	Joinpoint year (95% CI)	Trend segment 1: years, APC % (95% CI; p)	Trend segment 2: years, APC % (95% CI; p)	Years: AAPC, 1999–2024, % (95% CI; p)
Overall							
Overall (U.S., all ages)	16,113 (100.0)	0.21	0.19	2015 (2011–2018)	1999–2015: −1.75* (−2.53 to −0.97; p<0.001)	2015–2024: +3.07* (+1.25 to +4.93; p=0.002)	1999–2024: −0.04 (−0.81 to +0.73; p=0.917)
Sex							
Male	10,665 (66.2)	0.29	0.28	2015 (2013–2018)	1999–2015: −1.95* (−2.55 to −1.35; p<0.001)	2015–2024: +3.07* (+1.63 to +4.54; p<0.001)	1999–2024: −0.17 (−0.77 to +0.43; p=0.576)
Female	5,185 (32.2)	0.13	0.11	2015 (2006–2020)	1999–2015: −2.21* (−3.77 to −0.63; p=0.009)	2015–2024: +3.46* (+0.02 to +7.01; p=0.049)	1999–2024: −0.21 (−1.70 to +1.30; p=0.785)
Race and Hispanic origin							
Non-Hispanic White	13,883 (86.2)	0.22	0.24	2013 (2010–2017)	1999–2013: −1.41* (−2.29 to −0.52; p=0.003)	2013–2024: +3.16* (+1.91 to +4.43; p<0.001)	1999–2024: +0.58 (−0.11 to +1.27; p=0.102)
Non-Hispanic Black	1,086 (6.7)	0.14	0.08	None	1999–2024: −1.77* (−2.71 to −0.82; p<0.001)	—	1999–2024: −1.77* (−2.71 to −0.82; p<0.001)
Hispanic or Latino	661 (4.1)	0.10	0.06	None	2000–2024: −2.57* (−3.74 to −1.38; p<0.001)	—	2000–2024: −2.57* (−3.74 to −1.38; p<0.001)
U.S. Census region							
Northeast	2,964 (18.4)	0.21	0.19	None	1999–2024: −0.29 (−1.07 to +0.50; p=0.454)	—	1999–2024: −0.29 (−1.07 to +0.50; p=0.454)
Midwest	3,523 (21.9)	0.18	0.18	None	1999–2024: −0.09 (−0.68 to +0.50; p=0.749)	—	1999–2024: −0.09 (−0.68 to +0.50; p=0.749)
South	5,459 (33.9)	0.18	0.18	2014 (2010–2020)	1999–2014: −2.10* (−3.15 to −1.04; p<0.001)	2014–2024: +2.17* (+0.49 to +3.87; p=0.013)	1999–2024: −0.41 (−1.28 to +0.46; p=0.351)
West	4,167 (25.9)	0.25	0.20	2015 (2011–2019)	1999–2015: −1.75* (−2.85 to −0.63; p=0.004)	2015–2024: +3.13* (+0.78 to +5.54; p=0.011)	1999–2024: −0.02 (−1.05 to +1.03; p=0.975)

Trends by race and Hispanic origin

**Figure 3:**
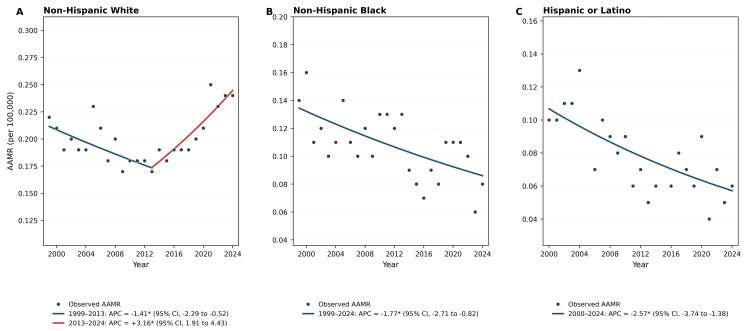
Age-adjusted mortality rates for hemochromatosis-related deaths by race and Hispanic origin, United States, 1999-2024. (A) Non-Hispanic White. (B) Non-Hispanic Black. (C) Hispanic or Latino. Non-Hispanic White mortality declined through 2013 (APC₁ = -1.41%, 95% CI, -2.29 to -0.52) and then rose significantly through 2024 (APC₂ = +3.16%, 95% CI, +1.91 to +4.43); AAPC for 1999-2024 was +0.58% (95% CI, -0.11 to +1.27), p = 0.102. Non-Hispanic Black mortality declined monotonically across the full period (APC = -1.77%, 95% CI, -2.71 to -0.82). Hispanic mortality declined monotonically across 2000-2024 (APC = -2.57%, 95% CI, -3.74 to -1.38); years 1999, 2005, and 2015 were excluded from modeling because CDC WONDER flagged the annual rate as unreliable (<20 deaths). *APC or AAPC significantly different from zero at α = 0.05 (95% CI excludes zero). Abbreviations: AAMR, age-adjusted mortality rate; APC, annual percent change; AAPC, average annual percent change; CI, confidence interval

Non-Hispanic White AAMR was 0.22 per 100,000 in 1999 and 0.24 per 100,000 in 2024 (Figure 3A; Table 2; annual fitted values, Appendix A). A single joinpoint was identified in 2013 (95% CI, 2010-2017), two years earlier than the overall joinpoint. AAMR declined at -1.41% per year (95% CI, -2.29 to -0.52; p = 0.003) from 1999 to 2013 and then rose at +3.16% per year (95% CI, +1.91 to +4.43; p < 0.001) from 2013 to 2024, with an AAPC of +0.58% (95% CI, -0.11 to +1.27; p = 0.102).

For non-Hispanic Black decedents, no joinpoint was identified; AAMR declined monotonically from 0.14 per 100,000 in 1999 to 0.08 per 100,000 in 2024, a 42.9% observed decrease (Figure 3B), with a single-segment AAPC of −1.77% (95% CI, −2.71 to −0.82; p < 0.001).

For Hispanic or Latino decedents, joinpoint regression was fit to 23 annual observations within the span 2000-2024 (1999, 2005, and 2015 excluded for CDC WONDER unreliable-rate flags; see Materials and Methods). No joinpoint was identified. AAMR declined monotonically from 0.10 per 100,000 in 2000 to 0.06 per 100,000 in 2024, a 40.0% observed decrease (Figure 3C), with a single-segment AAPC of −2.57% (95% CI, −3.74 to −1.38; p < 0.001) - the steepest sustained decline observed across any stratum in this analysis. Permutation-test details supporting the model selection in each racial and ethnic stratum are provided in Table 6.

The non-Hispanic White-to-non-Hispanic Black AAMR ratio widened from 1.57 in 1999 to 3.00 in 2024, and the non-Hispanic White-to-Hispanic ratio widened from 2.10 in 2000 to 4.00 in 2024. Non-Hispanic White decedents constituted the only racial or ethnic group in whom a post-joinpoint rise in AAMR was observed.

Trends by U.S. Census region

**Figure 4:**
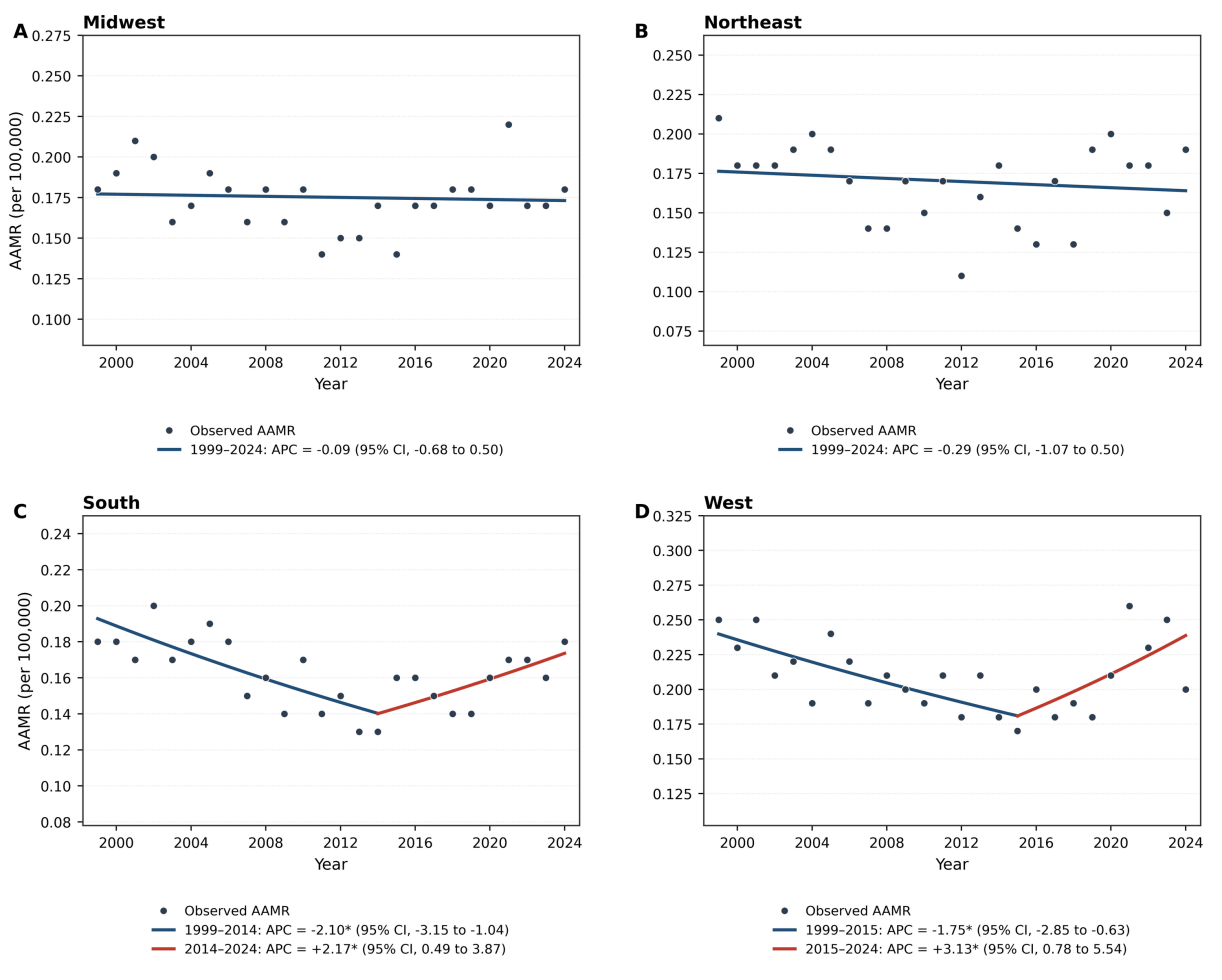
Age-adjusted mortality rates for hemochromatosis-related deaths by U.S. Census region, 1999-2024. (A) Midwest, (B) Northeast, (C) South, (D) West. Midwest (AAPC = -0.09%, p = 0.749) and Northeast (AAPC = -0.29%, p = 0.454) showed no significant change across 1999-2024. South and West both exhibited a declining APC, followed by a significant rebound: South Joinpoint 2014 (95% CI, 2010-2020), APC₁ = -2.10% and APC₂ = +2.17%; West Joinpoint 2015 (95% CI, 2011-2019), APC₁ = -1.75% and APC₂ = +3.13%. *APC significantly different from zero at α = 0.05. Abbreviations: APC, annual percent change; AAPC, average annual percent change; CI, confidence interval

In the Midwest, no joinpoint was identified; AAMR was 0.18 per 100,000 in both 1999 and 2024 (Figure 4A; Table 2), with a single-segment APC/AAPC of -0.09% (95% CI, -0.68 to +0.50; p = 0.749). In the Northeast, no joinpoint was identified; AAMR was 0.21 per 100,000 in 1999 and 0.19 per 100,000 in 2024 (Figure 4B), with an APC/AAPC of -0.29% (95% CI, -1.07 to +0.50; p = 0.454).

In the South, a single joinpoint was identified at 2014 (95% CI, 2010-2020). AAMR was 0.18 per 100,000 in 1999, declined to 0.13 per 100,000 in both 2013 and 2014, and recovered to 0.18 per 100,000 in 2024 (Figure 4C). Segment APCs were -2.10% (95% CI, -3.15 to -1.04; p < 0.001) for 1999-2014 and +2.17% (95% CI, +0.49 to +3.87; p = 0.013) for 2014-2024; the AAPC was -0.41% (95% CI, -1.28 to +0.46; p = 0.351).

In the West, a single joinpoint was identified in 2015 (95% CI, 2011-2019). AAMR was 0.25 per 100,000 in 1999, declined to 0.17 per 100,000 in 2015, and recovered to 0.20 per 100,000 in 2024 (Figure 4D). Segment APCs were -1.75% (95% CI, -2.85 to -0.63; p = 0.004) for 1999-2015 and +3.13% (95% CI, +0.78 to +5.54; p = 0.011) for 2015-2024; the AAPC was -0.02% (95% CI, -1.05 to +1.03; p = 0.975).

The two regions without a detected joinpoint (Midwest, Northeast) showed stable trajectories with no significant change across the full period. The two regions with joinpoints (South, West) reproduced the overall two-segment pattern, with the South's joinpoint (2014) preceding the West's (2015) by one year. Regional rank-order was broadly preserved: the West remained highest throughout (0.25 in 1999; 0.20 in 2024), while the Midwest and South remained the lowest-AAMR regions. Annual fitted regional AAMRs and the corresponding permutation-test sequences are tabulated in Appendix A and Appendix C, respectively.

Age-specific distribution and crude-rate gradient

Among the 15,703 age-classified deaths, the largest single stratum was 65-74 years (4,176 deaths; 26.6% of age-classified), followed by 75-84 years (3,870; 24.6%), 55-64 years (3,226; 20.5%), 45-54 years (1,725; 11.0%), and 85 years or older (1,660; 10.6%) (Table 1). Only 30 deaths occurred in decedents aged 15-24 years across the entire 26-year period.

Full-period crude mortality rates rose sharply with age, from 0.01 per 100,000 in the 15-24 year stratum to 1.15 per 100,000 in the 85-or-older stratum - a more than 80-fold gradient when computed from unrounded rates (1.148 vs. 0.014 per 100,000). Intermediate rates were 0.03 (25-34), 0.06 (35-44), 0.16 (45-54), 0.35 (55-64), 0.64 (65-74), and 1.05 (75-84) per 100,000 (Table 1).

Annual crude mortality in the 85-or-older stratum rose from 0.96 per 100,000 in 1999 to 1.76 per 100,000 in 2024, an 82.4% relative increase (computed from unrounded rates, 0.963 to 1.756 per 100,000), with annual death counts rising from 40 to 113. Rates in the 75-84 stratum remained comparatively stable (1.07 in 1999 to 1.13 in 2024), although annual death counts in this stratum rose from 131 to 219 (Appendix D).

State-level variation

Five states accounted for 5,194 deaths (32.2% of the national total): California (1,602), Florida (1,036), Texas (1,004), New York (785), and Pennsylvania (767) (Appendix E). The top 10 states - adding Ohio (667), North Carolina (533), Washington (533), Michigan (509), and Massachusetts (447) - accounted for 7,883 deaths (48.9%).

State-level AAMRs during 1999-2020 ranged from 0.07 per 100,000 (Mississippi) to 0.43 per 100,000 (Wyoming), a 6.1-fold range, with the five lowest-AAMR states being Mississippi (0.07), Louisiana (0.09), Georgia (0.10), and Illinois and New Jersey (each 0.11; New York was tied at 0.11), and the five highest being Wyoming (0.43), Rhode Island (0.41), and Oregon and Vermont (each 0.34), followed by Montana (0.30). During 2021-2024, AAMRs ranged from 0.08 per 100,000 (Louisiana and Mississippi) to 0.66 per 100,000 (Wyoming), an 8.3-fold range; the five highest-AAMR states were Wyoming (0.66), Colorado (0.48), Oregon (0.47), Vermont (0.43), and Idaho (0.42), and the five lowest were Louisiana and Mississippi (each 0.08), Illinois (0.09), Georgia (0.11), and New Jersey (0.12). Four jurisdictions (North Dakota, South Dakota, Delaware, District of Columbia) had fewer than 10 deaths during 2021-2024 and were suppressed in that file, accounting for the 29-death difference between combined state totals (16,084) and the overall national count (16,113). Full state-level deaths and AAMRs across both files are tabulated in Appendix E. State-level rates have wide confidence intervals for jurisdictions with small annual death counts; low-population states such as Wyoming are particularly susceptible to year-to-year volatility, and the highest-rate rankings should not be over-interpreted on the basis of point estimates alone.

Co-occurring causes of death

The frequency of co-listed conditions on E83.1-associated death certificates is summarized in Table 3. Chronic liver disease (K70-K76) was the most common co-listed condition, appearing on 7,412 certificates (46.0% of all 16,113 deaths); of these, 6,007 occurred during 1999-2020 and 1,405 during 2021-2024. Diabetes mellitus (E10-E14) was co-listed on 2,408 certificates (14.9%), primary liver cancer (C22) on 1,739 (10.8%), and viral hepatitis C (B17.1, B18.2) on 745 (4.6%). Heart failure (I50) appeared on 1,715 certificates (10.6%) and cardiomyopathy (I42) on 674 (4.2%). Sepsis (A40-A41) was co-listed on 1,193 certificates (7.4%) and gout (M10) on 50 (0.3%). Because a single certificate may list more than one co-occurring condition, individual decedents may be represented across multiple rows; the eight conditions therefore do not sum to the overall total.

**Table 3:** Co-occurring causes of death listed with hemochromatosis (ICD-10 E83.1) on U.S. death certificates, 1999-2024 Each row reports deaths with ICD-10 code E83.1 and the listed co-occurring cause both present on the death certificate (underlying or contributing cause), extracted from the Centers for Disease Control and Prevention (CDC) Wide-ranging Online Data for Epidemiologic Research (WONDER) Multiple Cause of Death files. Conditions are shown in descending order of co-occurrence frequency. Rows do not sum to 16,113 because a single death certificate may list more than one co-occurring condition. Percentages are of the overall 16,113 hemochromatosis-related deaths during 1999-2024. Counts for conditions with most annual cells <10 (e.g., gout) use the CDC WONDER's aggregate "Total" row, which is computed from unsuppressed data. Crude rate per 100,000 U.S. population computed as total co-occurrence deaths divided by total population-years during the relevant observation period; 95% CI by normal approximation.

Co-occurring condition	ICD-10 code(s)	Deaths co-listing both conditions, N (% of 16,113)	Crude rate per 100,000 (95% CI)	Deaths, 1999–2020	Deaths, 2021-2024
Chronic liver disease	K70–K76	7,412 (46.0)	0.092 (0.090-0.094)	6,007	1,405
Diabetes mellitus	E10–E14	2,408 (14.9)	0.030 (0.029-0.031)	1,904	504
Liver cancer (hepatocellular)	C22	1,739 (10.8)	0.022 (0.020-0.023)	1,416	323
Heart failure	I50	1,715 (10.6)	0.021 (0.020-0.022)	1,338	377
Sepsis	A40–A41	1,193 (7.4)	0.015 (0.014-0.016)	964	229
Viral hepatitis C	B17.1, B18.2	745 (4.6)	0.009 (0.009-0.010)	657	88
Cardiomyopathy	I42	674 (4.2)	0.008 (0.008-0.009)	584	90
Gout	M10	50 (0.3)	0.001 (0.000-0.001)	35	15

## Discussion

Summary of principal findings

Building on the foundational work of Yang and colleagues (1979-1992) [12] and Brown and colleagues (1979-1997) [13], we present a 26-year analysis covering the subsequent period (1999-2024). From 1999 through 2024, we identified 16,113 U.S. deaths with hemochromatosis (ICD-10 E83.1) listed as an underlying or contributing cause. Three findings stand out. First, the full-period AAPC was essentially flat (-0.04%, p = 0.917) yet concealed a two-segment trajectory: a 16-year decline of -1.75% per year (p < 0.001), followed by a nine-year rebound of +3.07% per year (p = 0.002), with a joinpoint at 2015 (95% CI, 2011-2018). Second, the burden was demographically concentrated - men 66.2%, non-Hispanic White decedents 86.2%, and adults aged 65 years and older 61.8% of age-classified deaths. Third, within the stratified racial and ethnic analysis, a post-joinpoint rebound was observed only among non-Hispanic White decedents; non-Hispanic Black and Hispanic rates declined monotonically throughout. The comorbidity profile reinforced the classical end-organ pattern of iron overload, with chronic liver disease (K70-K76) listed on 46.0% of death certificates and primary liver cancer (C22) on 10.8%, alongside diabetes mellitus, heart failure, cardiomyopathy, and sepsis.

2013-2015 inflection and net-flat AAPC

As set out in the Introduction, ICD-10 E83.1 is not genotype-specific; we cannot separate HFE-related hereditary disease from non-HFE hereditary and secondary forms from aggregate death-certificate data. The interpretation that follows therefore draws selective inference from HFE-focused cohort studies and flags the primary-versus-secondary distinction at each point where it materially affects interpretation.

An AAPC not significantly different from zero can be misread as stability; our segments indicate the opposite. The 33.3% cumulative decline through 2015 was nearly offset by a 35.7% cumulative rebound through 2024, a pattern reproduced in male, female, South, and West subgroups. Mid-decade inflections have become a recurring feature of the CDC WONDER-based U.S. mortality analyses, and whether these inflections share a common origin warrants examination. Bhagwan and colleagues, studying sudden cardiac death across 1999-2020 (n = 279,599), reported an inflection in 2018 with a subsequent APC of +6.93% across 2018-2020 [15]. Tuin and colleagues described a reversal in hemolytic-anemia mortality (1999-2022, n = 46,924) with an overall inflection at 2016 [16], and Ahmed and colleagues documented rising amyloidosis-cardiovascular-disease co-coded mortality across 1999-2020 (n = 26,391) [17]. A uniform coding or surveillance-platform artifact would be expected to raise mortality rates across all racial and ethnic groups roughly in proportion to their baseline disease prevalence. In our data, the post-2013 rebound was confined to non-Hispanic White decedents, while non-Hispanic Black and Hispanic rates declined monotonically across the full study period (see Race and Hispanic-origin disparities below). This demographic specificity is more consistent with disease-specific biology than with a uniform coding shift across heterogeneous chronic conditions, and points toward a process operating selectively on the HFE-harboring population. The 2015 joinpoint also precedes the COVID-19 pandemic by several years, ruling out pandemic initiation of the reversal; the rebound segment did encompass the pandemic interval, with a rate peak in 2021 (0.21 per 100,000) followed by partial decline to 0.19 by 2024 - a pattern consistent with, but not proof of, a transient pandemic-era contribution superimposed on a pre-existing trajectory. The methodological implication is that segment-specific APCs, rather than AAPCs, carry the meaningful signal when a joinpoint is present.

A second consideration concerns the interpretation of the chronic-liver-disease overlap. ICD-10 E83.1 captures all disorders of iron metabolism, including secondary iron overload arising from cirrhosis of any etiology. The 46.0% co-occurrence with chronic liver disease (K70-K76) is therefore consistent with two non-mutually exclusive scenarios: deaths from primary HFE-related hemochromatosis that progressed to cirrhosis, and deaths from cirrhosis of independent etiology (alcohol-related, viral hepatitis-related, or metabolic) in which secondary iron loading led to E83.1 being coded on the death certificate. These cannot be separated from aggregate death certificate data. The demographic concentration of E83.1-coded deaths in older non-Hispanic White men is more consistent with primary HFE-related disease - the demographic group in which C282Y homozygosity is concentrated, but the chronic-liver-disease overlap leaves a substantial proportion ambiguous. This bounds the inference that the post-2013 rebound represents undertreated HFE disease specifically, rather than evolving cirrhosis epidemiology more broadly.

Sex disparities

Mortality was male-predominant throughout, and the male-to-female ratio widened from 2.23 in 1999 to 2.55 in 2024. Both sexes followed parallel two-segment trajectories (male Seg 1 -1.95%, Seg 2 +3.07%; female Seg 1 -2.21%, Seg 2 +3.46%). The female post-joinpoint slope reached only nominal significance (p = 0.049; CI lower bound +0.02), so the female rebound is interpreted cautiously: directional agreement with the male trajectory is consistent with a common underlying process, although the female estimate alone is fragile and could shift meaningfully under alternative model specifications or with longer follow-up. The sex gradient is anchored in iron physiology - menstruation and childbirth attenuate premenopausal iron accumulation. In HFE-genotyped populations, these differences translate into substantial penetrance disparities. Direct U.S. genotype-to-mortality estimates are not available; UK Biobank estimates are used here as the best contemporaneous quantitative anchor, with the generalizability limits noted in the Introduction. Pilling and colleagues, in the UK Biobank, reported that among 2,890 p.C282Y homozygotes, hemochromatosis was diagnosed during follow-up in 21.7% of men versus 9.8% of women [8]. With longer follow-up, Lucas and colleagues found that by age 80 the cumulative incidence of liver disease reached 20.3% in male homozygotes (versus 8.3% in men without HFE variants) and documented excess all-cause mortality in homozygous men (hazard ratio 1.29; 95% CI, 1.12-1.48); in women, cumulative liver-disease incidence was 8.9% versus 6.8% and mortality was not elevated [9]. Atkins and colleagues, in the same cohort, reported a hazard ratio of 10.5 (95% CI, 6.6-16.7) for primary hepatic malignancy in male homozygotes, with no significant homozygosity-associated risks in women [10]. Assuming HFE disease accounts for most E83.1 deaths, these penetrance differences are consistent with the widening male-to-female mortality ratio we observed, and suggest the post-2015 rebound has been driven disproportionately by male decedents.

Race and Hispanic-origin disparities

Within stratified racial and ethnic analysis, non-Hispanic White decedents were the only group in whom a post-joinpoint rebound occurred: the rate fell from 0.22 per 100,000 in 1999 to a 2013 nadir of 0.17 and rose to 0.24 in 2024 (Seg 2 +3.16%, p < 0.001). The full-period AAPC of +0.58% did not reach significance (p = 0.102) because the preceding decline partly offset the rise. Non-Hispanic Black and Hispanic rates declined monotonically (AAPCs −1.77% and −2.57%, both p < 0.001). The non-Hispanic White:non-Hispanic Black ratio grew from 1.57 to 3.00, and the non-Hispanic White:Hispanic ratio from 2.10 to 4.00. This pattern is congruent with the founder origin of the HFE p.C282Y variant. Hanson and colleagues' HuGE review documented a heterozygote frequency of approximately 9.2% in European populations and essentially none in African, Asian, and most Indigenous populations [6]. The HEIRS study quantified the disparity in a U.S. primary-care sample of nearly 100,000 adults: C282Y homozygote prevalence was 4.4 per 1,000 in non-Hispanic Whites, 0.27 per 1,000 in Hispanics, and 0.14 per 1,000 in African Americans - a 31-fold non-Hispanic White:non-Hispanic Black gradient and a 16-fold non-Hispanic White:Hispanic gradient [7]. We interpret the simultaneous rise in non-Hispanic White mortality and monotonic decline in non-Hispanic Black and Hispanic mortality as more consistent with a penetrance- or ascertainment-mediated mechanism acting specifically on the HFE-harboring population than with a uniform coding-improvement mechanism, which would be expected to raise recorded mortality across groups in proportion to baseline disease prevalence. Alternative explanations cannot be excluded from mortality data alone; discriminating among penetrance, recognition, and coding would require individual-level longitudinal data linking HFE genotype, treatment exposure, and outcomes. The Hispanic decline is the steepest in our dataset, but several factors complicate interpretation: endpoint counts are small (three years required exclusion for unreliable rates), age-structure shifts within the U.S. Hispanic population during 2000-2024 may not be fully captured by direct age standardization to the 2000 standard, and case-ascertainment differences between the bridged-race and single-race files may differentially affect the Hispanic stratum where C282Y prevalence is genuinely low. The Hispanic AAPC point estimate should accordingly be interpreted as directional rather than precise. A similar racial signature has been reported in other CDC WONDER analyses of liver-disease endpoints, including Abboud and colleagues on hepatocellular carcinoma [24].

Regional and urbanization disparities

Only the South and West showed joinpoint-confirmed two-segment trajectories, with inflections at 2014 (95% CI, 2010-2020) and 2015 (95% CI, 2011-2019); no inflection was detected in the Northeast or Midwest. The West remained the highest-mortality region throughout (0.25 per 100,000 in 1999; 0.20 in 2024), while the Midwest and South had the lowest regional rates at both endpoints - broadly consistent with Northern-European-ancestry settlement patterns. State-level rates spanned 6.1-fold in 1999-2020 (Mississippi 0.07 to Wyoming 0.43) and 8.3-fold in 2021-2024 (Louisiana and Mississippi 0.08 to Wyoming 0.66); Wyoming, Colorado, Oregon, Vermont, and Idaho consistently ranked the highest. Analyses of other chronic conditions - Kwaah and colleagues on chronic ischemic heart disease [25], Yasmin and colleagues on cardiovascular disease with coexisting liver cirrhosis [26] - suggest rural-urban gradients tend to widen over time. Formal rural-urban trend analysis for hemochromatosis was not feasible in the 2021-2024 data because the CDC WONDER single-race urbanization export does not include population denominators.

Age gradient and the aging U.S. population

The age gradient was steep: the full-period crude mortality rate in the 85-and-older stratum (1.148 per 100,000) exceeded that in the 15-24-year stratum (0.014) by more than 80-fold. The 85-and-older stratum showed the largest single-stratum change, with crude mortality rising from 0.96 per 100,000 in 1999 to 1.76 in 2024 (+82.4% from unrounded rates) and annual deaths nearly tripling from 40 to 113. This increase reflects both an absolute rise in deaths and changes in the size and age structure of the 85-and-older population over the period. In the 75-84-year stratum, rates were comparatively stable (1.07 to 1.13), but absolute deaths rose from 131 to 219 per year, reflecting the impact of population aging on absolute burden even when age-specific rates do not change. These patterns fit the biology of hemochromatosis as a disease of cumulative iron deposition. Grosse and colleagues estimated that roughly one in ten male C282Y homozygotes will develop severe liver disease over a lifetime if iron overload is not detected and treated early [27]; Lucas and colleagues placed cumulative liver-disease incidence at 20.3% by age 80 in male homozygotes [9].

Comorbidity profile and end-organ pathophysiology

The frequencies at which selected end-organ conditions co-appeared on E83.1-coded death certificates are interpretable in light of established hemochromatosis pathophysiology. Chronic liver disease (46.0%) and primary liver cancer (10.8%) are the dominant terminal endpoints of unchecked hepatic iron deposition, with the relative risk of hepatocellular carcinoma in established cirrhosis approximately 20-fold and an annual incidence of 3-4% in untreated disease [3,4]. Diabetes mellitus (14.9%) reflects the pancreatic toxicity of iron overload: deposition is largely restricted to islet beta-cells, and the resulting loss of insulin secretory capacity - termed "bronze diabetes" in the original 19th-century descriptions - has been a sine qua non of the historical hemochromatosis phenotype [1-3]. Heart failure (10.6%) and cardiomyopathy (4.2%) reflect the cardiac trajectory of iron overload, in which myocardial iron deposition typically presents first as restrictive dysfunction and conduction abnormality before evolving to dilated cardiomyopathy and overt failure [1,2]; the higher proportion of heart failure than cardiomyopathy in our data is therefore biologically expected, with cardiomyopathy capturing the later-stage cases predominantly. Sepsis (7.4%) is consistent with the well-established susceptibility of iron-overloaded hosts to siderophilic bacteria - particularly Vibrio vulnificus and Yersinia species - which exploit the elevated non-transferrin-bound iron and impaired hepcidin response characteristic of HFE disease [28]. Hepatitis C (4.6%) likely reflects two non-exclusive scenarios in our population: HCV-associated cirrhosis as a secondary cause of hepatic iron loading triggering E83.1 coding, and HCV co-infection in patients with primary HFE disease, since HCV suppresses hepcidin transcription and thereby aggravates iron accumulation [2]. Gout (0.3%) is captured but interpretively limited because most year-level cells were suppressed; the historical association between hemochromatosis and arthropathy includes urate-related joint disease as a recognized but uncommon endpoint [3,4]. The rank order of these conditions - liver disease and liver neoplasms first, followed by metabolic and cardiac sequelae - mirrors the proportionate-mortality pattern reported by Yang and colleagues for the 1979-1992 period [12], suggesting that the underlying pathophysiology of E83.1-coded mortality has not changed materially across the post-ICD-10 era.

Clinical and public health implications

A post-2013 rise in mortality within the non-Hispanic White stratum, for a condition with effective, low-cost treatment (therapeutic phlebotomy), warrants careful examination. Three non-exclusive mechanisms merit consideration; their relative contributions cannot be separated from aggregate mortality data. First, improved recognition and more complete death-certificate coding likely contribute. Abou Yassine and colleagues observed a 79% rise in hemochromatosis-related hospitalizations between 2002 and 2010 (34.5 to 61.4 per 100,000 hospitalizations) in the Nationwide Inpatient Sample [14]. This hospitalization increase occurred during the decline phase of our mortality series (1999-2015); divergent directions are informative, being consistent with either decreasing case-fatality (improved treatment) or ascertainment of milder cases expanding faster than case-fatality was declining. Second, cohort effects may contribute: homozygous individuals reaching ages of

Clinical sequelae during the post-2013 period being predominantly birth cohorts

From before 1960, born in an era when HFE genotyping was not available and when clinical recognition was more limited, these cohorts may now be presenting with end-organ disease that, in younger cohorts diagnosed since the early 2000s, could have been prevented by earlier treatment. This framing aligns with our 82.4% increase in the 85-and-older crude rate and the near-tripling of absolute deaths in that stratum. Third, undertreatment or delayed treatment in recognized cases remains a concern; Niederau and colleagues established three decades ago that patients diagnosed before cirrhosis and diabetes develop can achieve normal life expectancy with treatment, yet real-world adherence and specialty access vary [11]. The comorbidity profile - chronic liver disease 46.0%, diabetes 14.9%, heart failure 10.6%, liver cancer 10.8%, sepsis 7.4%, hepatitis C 4.6%, cardiomyopathy 4.2% - remains dominated by the same end-organ targets identified by Yang and colleagues (liver disease, liver neoplasms, cardiomyopathy) [12], though direct numerical comparison with their proportionate mortality ratios is not possible from our descriptive proportions, which use a different denominator. The stability of this end-organ pattern across 26 years suggests that the underlying pathophysiology has not changed even as recognition has shifted. Our findings do not overturn the existing screening framework. The 2006 USPSTF recommendation against routine population screening [29] was based on low clinical penetrance, potential harms of genetic labeling, and the absence of randomized-trial evidence that screening improves outcomes. The USPSTF has subsequently classified hemochromatosis screening as an Inactive topic and has stated it will not review or update this recommendation [30]; no updated U.S. screening guidance has been issued. The absolute hemochromatosis-associated mortality we document (approximately 0.19 per 100,000 in 2024) remains small in population terms. Subsequent UK Biobank evidence has documented excess all-cause mortality in male C282Y homozygotes, a hazard ratio of 10.5 for primary hepatic malignancy, and non-trivial cumulative liver-disease incidence by age 80 [9,10,27]. Our findings contribute population-level mortality data alongside this UK Biobank evidence that may inform future evidence reviews if this topic is reactivated, particularly in the demographic subgroup where the post-2013 rebound has been concentrated.

Strengths and limitations

Strengths include full national coverage across all 50 states and the District of Columbia over 26 years (n = 16,113 deaths) with no sampling uncertainty at the ascertained level; use of the Multiple Cause of Death file, an approach Minhas and colleagues argue is essential because underlying-cause-only analyses materially underestimate burden [18]; a pre-specified NCI Joinpoint analysis with Monte Carlo permutation testing (Kim and colleagues) [19] enabling direct comparison with the CDC WONDER trend literature; transparent handling of cell-suppression rules; and compliance with STROBE and RECORD standards [20,21]. Several limitations warrant emphasis. ICD-10 E83.1 does not distinguish primary HFE-related hemochromatosis from non-HFE hereditary forms or secondary iron overload, and our dataset carries no clinical information on HFE genotype, treatment exposure, or ferritin trajectories; genotype-to-outcome inferences drawn from UK Biobank must therefore be regarded as contextual rather than direct validation. Death-certificate coding is subject to variable physician awareness and miscoding, and hemochromatosis has historically been under-recognized [1,2]; trend estimates cannot separate changes in underlying occurrence from changes in recognition or coding. The NCHS transition from bridged-race (1999-2020) to single-race (2021-2024) classification introduces a discontinuity we minimized by restricting stratified trend analyses to the bridged-race period; for the overall AAMR series, however, the file boundary at 2020/2021 falls within the rebound segment identified by joinpoint, and a residual contribution of the file transition to the post-2015 rebound cannot be excluded from the headline analysis alone. Cell suppression of counts below 10 removed 263 deaths from sex-stratified analyses, 410 from age-stratified analyses, and 29 from the state-level total; three years of Hispanic-origin data (1999, 2005, 2015) were excluded for unreliable rates, and the Hispanic AAPC should accordingly be considered the most fragile of our stratified estimates. The 2021-2024 urbanization export lacked population denominators, precluding stratified rate calculation for that period. We did not perform sensitivity analyses using alternative joinpoint model-selection criteria (e.g., Hudson's method or BIC); these would not be expected to change qualitative direction, but may shift joinpoint placement by one to two years. International comparisons with Danish, Swedish, and Australian hemochromatosis cohort and registry data were outside our scope but would strengthen contextual interpretation in future work. Finally, the analysis is descriptive; trend reversals cannot be attributed causally to specific events from mortality data alone.

## Conclusions

This 26-year nationwide analysis of hemochromatosis-associated mortality in the United States identified a statistically significant trend reversal in 2015, with a 16-year decline followed by a nine-year rebound that was concentrated in older non-Hispanic White men. Non-Hispanic Black and Hispanic mortality declined monotonically throughout the period, widening race and ethnic disparities. The rank order and approximate proportions of co-occurring end-organ conditions on hemochromatosis-coded death certificates remained stable across the observation period, indicating that the clinical signature of hemochromatosis-associated mortality has not shifted materially. These findings highlight a concentrated burden of hemochromatosis-associated mortality among older non-Hispanic White men, particularly during the post-2013 period. Distinguishing coding, cohort, and penetrance contributions to the observed rebound will require individual-level data linking HFE genotype, treatment exposure, and long-term clinical outcomes.

## Appendices

Appendix A 

**Table 4:** Annual observed age-adjusted mortality rate, joinpoint-fitted AAMR, and standard error for hemochromatosis-related deaths (ICD-10 E83.1), United States, 1999–2024, by stratum. Observed AAMRs and standard errors are from CDC WONDER age-adjusted rate output (reported to two decimals; 2000 U.S. standard population). Fitted values computed as exp(intercept + slope × year) using segment-specific parameters from the Joinpoint Regression Program v6.0.1 [19]. Hispanic years 1999, 2005, and 2015 were excluded from modeling because CDC WONDER flagged the annual rate as unreliable (<20 deaths). Strata include overall, sex, race, and Hispanic origin, and U.S. Census region. Abbreviations: AAMR, age-adjusted mortality rate; SE, standard error; CDC, Centers for Disease Control and Prevention; ICD-10, International Classification of Diseases, 10th Revision

Year	Overall (U.S., all ages)			Male			Female			Non-Hispanic White			Non-Hispanic Black			Hispanic or Latino			Northeast			Midwest			South			West		
	Observed	Fitted	SE	Observed	Fitted	SE	Observed	Fitted	SE	Observed	Fitted	SE	Observed	Fitted	SE	Observed	Fitted	SE	Observed	Fitted	SE	Observed	Fitted	SE	Observed	Fitted	SE	Observed	Fitted	SE
1999	0.21	0.1993	0.0077	0.29	0.3068	0.0153	0.13	0.1226	0.0102	0.22	0.2115	0.0102	0.14	0.1345	0.0230	—	—	—	0.21	0.1763	0.0204	0.18	0.1772	0.0153	0.18	0.1928	0.0153	0.25	0.2398	0.0230
2000	0.20	0.1958	0.0102	0.28	0.3008	0.0153	0.12	0.1199	0.0077	0.21	0.2085	0.0102	0.16	0.1321	0.0230	0.10	0.1067	0.0281	0.18	0.1758	0.0179	0.19	0.1770	0.0179	0.18	0.1888	0.0128	0.23	0.2357	0.0204
2001	0.19	0.1923	0.0077	0.28	0.2949	0.0153	0.13	0.1172	0.0077	0.19	0.2056	0.0077	0.11	0.1298	0.0179	0.10	0.1040	0.0281	0.18	0.1753	0.0179	0.21	0.1769	0.0179	0.17	0.1848	0.0153	0.25	0.2315	0.0204
2002	0.18	0.1890	0.0077	0.31	0.2892	0.0153	0.10	0.1146	0.0077	0.20	0.2027	0.0102	0.12	0.1275	0.0230	0.11	0.1013	0.0306	0.18	0.1748	0.0179	0.20	0.1767	0.0204	0.20	0.1809	0.0153	0.21	0.2275	0.0179
2003	0.17	0.1857	0.0077	0.27	0.2835	0.0153	0.09	0.1121	0.0077	0.19	0.1998	0.0077	0.10	0.1252	0.0179	0.11	0.0987	0.0281	0.19	0.1743	0.0179	0.16	0.1766	0.0179	0.17	0.1771	0.0153	0.22	0.2235	0.0204
2004	0.18	0.1824	0.0077	0.28	0.2780	0.0153	0.11	0.1096	0.0077	0.19	0.1970	0.0102	0.11	0.1230	0.0230	0.13	0.0962	0.0306	0.20	0.1738	0.0179	0.17	0.1764	0.0153	0.18	0.1734	0.0128	0.19	0.2196	0.0179
2005	0.20	0.1792	0.0077	0.30	0.2726	0.0153	0.13	0.1072	0.0102	0.23	0.1942	0.0102	0.14	0.1208	0.0230	—	—	—	0.19	0.1733	0.0204	0.19	0.1762	0.0179	0.19	0.1698	0.0128	0.24	0.2158	0.0204
2006	0.17	0.1761	0.0077	0.27	0.2673	0.0153	0.10	0.1048	0.0077	0.21	0.1915	0.0102	0.11	0.1187	0.0204	0.07	0.0913	0.0204	0.17	0.1728	0.0179	0.18	0.1761	0.0153	0.18	0.1662	0.0128	0.22	0.2120	0.0204
2007	0.15	0.1730	0.0077	0.26	0.2620	0.0153	0.08	0.1025	0.0077	0.18	0.1888	0.0077	0.10	0.1166	0.0179	0.10	0.0890	0.0255	0.14	0.1723	0.0153	0.16	0.1759	0.0153	0.15	0.1627	0.0102	0.19	0.2083	0.0179
2008	0.18	0.1700	0.0077	0.28	0.2569	0.0153	0.11	0.1002	0.0077	0.20	0.1861	0.0102	0.12	0.1145	0.0204	0.09	0.0867	0.0230	0.14	0.1718	0.0153	0.18	0.1757	0.0153	0.16	0.1593	0.0102	0.21	0.2047	0.0179
2009	0.16	0.1670	0.0051	0.27	0.2519	0.0153	0.08	0.0980	0.0077	0.17	0.1835	0.0077	0.10	0.1125	0.0179	0.08	0.0844	0.0179	0.17	0.1713	0.0153	0.16	0.1756	0.0153	0.14	0.1560	0.0102	0.20	0.2011	0.0153
2010	0.17	0.1641	0.0077	0.26	0.2470	0.0128	0.10	0.0958	0.0077	0.18	0.1809	0.0077	0.13	0.1105	0.0204	0.09	0.0823	0.0204	0.15	0.1708	0.0153	0.18	0.1754	0.0179	0.17	0.1527	0.0128	0.19	0.1976	0.0179
2011	0.16	0.1612	0.0077	0.24	0.2422	0.0128	0.10	0.0937	0.0077	0.18	0.1784	0.0077	0.13	0.1086	0.0204	0.06	0.0802	0.0153	0.17	0.1703	0.0153	0.14	0.1753	0.0128	0.14	0.1495	0.0128	0.21	0.1941	0.0179
2012	0.16	0.1584	0.0077	0.22	0.2374	0.0128	0.08	0.0916	0.0077	0.18	0.1759	0.0077	0.12	0.1066	0.0204	0.07	0.0781	0.0179	0.11	0.1698	0.0128	0.15	0.1751	0.0153	0.15	0.1464	0.0128	0.18	0.1908	0.0153
2013	0.16	0.1556	0.0077	0.23	0.2328	0.0102	0.10	0.0896	0.0102	0.17	0.1734	0.0077	0.13	0.1047	0.0204	0.05	0.0761	0.0153	0.16	0.1693	0.0153	0.15	0.1749	0.0153	0.13	0.1433	0.0102	0.21	0.1874	0.0179
2014	0.16	0.1529	0.0051	0.23	0.2283	0.0128	0.09	0.0876	0.0077	0.19	0.1792	0.0102	0.09	0.1029	0.0179	0.06	0.0741	0.0153	0.18	0.1688	0.0179	0.17	0.1748	0.0153	0.13	0.1403	0.0102	0.18	0.1842	0.0153
2015	0.14	0.1502	0.0051	0.21	0.2238	0.0102	0.09	0.0857	0.0077	0.18	0.1849	0.0077	0.08	0.1011	0.0153	—	—	—	0.14	0.1683	0.0153	0.14	0.1746	0.0153	0.16	0.1431	0.0102	0.17	0.1809	0.0153
2016	0.16	0.1548	0.0077	0.23	0.2307	0.0128	0.09	0.0886	0.0051	0.19	0.1908	0.0102	0.07	0.0993	0.0128	0.06	0.0704	0.0153	0.13	0.1678	0.0153	0.17	0.1744	0.0128	0.16	0.1462	0.0128	0.20	0.1865	0.0153
2017	0.16	0.1595	0.0077	0.23	0.2378	0.0128	0.08	0.0917	0.0077	0.19	0.1968	0.0102	0.09	0.0975	0.0153	0.08	0.0686	0.0179	0.17	0.1673	0.0153	0.17	0.1743	0.0153	0.15	0.1494	0.0102	0.18	0.1923	0.0128
2018	0.15	0.1644	0.0077	0.25	0.2451	0.0128	0.09	0.0949	0.0077	0.19	0.2030	0.0102	0.08	0.0958	0.0128	0.07	0.0668	0.0153	0.13	0.1668	0.0153	0.18	0.1741	0.0153	0.14	0.1526	0.0102	0.19	0.1984	0.0153
2019	0.16	0.1695	0.0051	0.24	0.2526	0.0102	0.07	0.0981	0.0077	0.20	0.2094	0.0077	0.11	0.0941	0.0179	0.06	0.0651	0.0153	0.19	0.1664	0.0179	0.18	0.1740	0.0153	0.14	0.1559	0.0102	0.18	0.2046	0.0153
2020	0.19	0.1747	0.0077	0.26	0.2604	0.0128	0.11	0.1015	0.0077	0.21	0.2161	0.0102	0.11	0.0924	0.0179	0.09	0.0634	0.0179	0.20	0.1659	0.0179	0.17	0.1738	0.0153	0.16	0.1593	0.0128	0.21	0.2110	0.0153
2021	0.21	0.1801	0.0077	0.29	0.2684	0.0128	0.14	0.1051	0.0102	0.25	0.2229	0.0102	0.11	0.0908	0.0179	0.04	0.0618	0.0128	0.18	0.1654	0.0179	0.22	0.1736	0.0153	0.17	0.1628	0.0128	0.26	0.2176	0.0179
2022	0.19	0.1856	0.0077	0.28	0.2766	0.0128	0.11	0.1087	0.0077	0.23	0.2299	0.0077	0.10	0.0892	0.0153	0.07	0.0602	0.0153	0.18	0.1649	0.0179	0.17	0.1735	0.0153	0.17	0.1663	0.0128	0.23	0.2244	0.0153
2023	0.18	0.1913	0.0077	0.29	0.2851	0.0153	0.10	0.1125	0.0077	0.24	0.2372	0.0102	0.06	0.0876	0.0128	0.05	0.0587	0.0128	0.15	0.1644	0.0153	0.17	0.1733	0.0153	0.16	0.1699	0.0128	0.25	0.2315	0.0179
2024	0.19	0.1972	0.0051	0.28	0.2939	0.0128	0.11	0.1163	0.0077	0.24	0.2447	0.0077	0.08	0.0860	0.0153	0.06	0.0572	0.0128	0.19	0.1640	0.0153	0.18	0.1732	0.0153	0.18	0.1736	0.0102	0.20	0.2387	0.0153

Appendix B 

**Table 5:** Joinpoint regression: final selected model and segment parameters per stratum Final selected joinpoint model for each analytic stratum, including number of joinpoints, joinpoint year(s) with 95% CI, number of observations (N), model degrees of freedom (DF), residual sum of squared errors (SSE), mean squared error (MSE), and segment-specific intercepts (β₀) and slopes (β₁). Joinpoint regression fit using the NCI Joinpoint Regression Program v6.0.1 [19]; log-linear model; uncorrelated errors; grid search; minimum two observations between joinpoints and between any joinpoint and the series endpoints. *Joinpoint year identified by the Monte Carlo permutation test as significantly different from the no-joinpoint null (p < α; selection sequence shown in Table 6). Abbreviations: JP, joinpoint; Seg, segment; CI, confidence interval; DF, degrees of freedom; SSE, sum of squared errors; MSE, mean squared error

Stratum	Final model (# joinpoints)	Joinpoint year(s)	N obs.	# parameters	Residual DF	SSE	MSE	Segment	Intercept	Slope (log-rate/yr)	APC %
Overall (U.S., all ages)	1	2015 (2011–2018)	26	4	22	67.4190	3.0645	Seg 0: 1999–2015	33.7152	-0.017673	−1.75*
								Seg 1: 2015–2024	-62.9042	0.030277	+3.07*
Male	1	2015 (2013–2018)	26	4	22	25.0585	1.1390	Seg 0: 1999–2015	38.2227	-0.019712	−1.95*
								Seg 1: 2015–2024	-62.4486	0.030249	+3.07*
Female	1	2015 (2006–2020)	26	4	22	68.9302	3.1332	Seg 0: 1999–2015	42.6587	-0.022390	−2.21*
								Seg 1: 2015–2024	-70.9794	0.034006	+3.46*
Non-Hispanic White	1	2013 (2010–2017)	26	4	22	44.9551	2.0434	Seg 0: 1999–2013	26.8063	-0.014187	−1.41*
								Seg 1: 2013–2024	-64.4512	0.031148	+3.16*
Non-Hispanic Black	0	None	26	2	24	26.2126	1.0922	Seg 0: 1999–2024	33.7302	-0.017877	−1.77*
Hispanic or Latino	0	None	23	2	21	14.1777	0.6751	Seg 0: 2000–2024	49.8025	-0.026020	−2.57*
Northeast	0	None	26	2	24	55.0148	2.2923	Seg 0: 1999–2024	4.0655	-0.002902	−0.29
Midwest	0	None	26	2	24	37.0794	1.5450	Seg 0: 1999–2024	0.1247	-0.000928	−0.09
South	1	2014 (2010–2020)	26	4	22	26.2200	1.1918	Seg 0: 1999–2014	40.7467	-0.021207	−2.10*
								Seg 1: 2014–2024	-45.1436	0.021439	+2.17*
West	1	2015 (2011–2019)	26	4	22	28.6223	1.3010	Seg 0: 1999–2015	33.7786	-0.017612	−1.75*
								Seg 1: 2015–2024	-63.8689	0.030848	+3.13*

Appendix C

**Table 6:** Joinpoint regression: Monte Carlo permutation test sequence per stratum Monte Carlo permutation test sequence used to select the optimal number of joinpoints in each stratum. Each row reports the test number, null and alternative joinpoint counts (H₀ and H₁), the number selected, numerator degrees of freedom (DF₁), denominator degrees of freedom (DF₂), number of permutations (4,499 per test), p-value, and the per-test significance threshold. Optimal joinpoint number selected as described by Kim and colleagues [19]; overall significance level α = 0.05. *Permutation test selected as the final accepted model for the stratum (see corresponding model parameters in Table 5). Abbreviations: H₀, null hypothesis; H₁, alternative hypothesis; DF, degrees of freedom

Stratum	Test #	H₀ (# JPs)	H₁ (# JPs)	Selected	Num. DF	Den. DF	# permutations	P-value	Signif. level
Overall (U.S., all ages)	0	0	4	4	8	16	4500	0.0002222	0.0125000
	1	1	4	1	6	16	4500	0.1377778	0.0166667
	2	1	3	1	4	18	4500	0.0675556	0.0166667
	3	1	2	1	2	20	4500	0.0346667	0.0166667
Male	0	0	4	4	8	16	4500	0.0002222	0.0125000
	1	1	4	1	6	16	4500	0.0615556	0.0166667
	2	1	3	1	4	18	4500	0.0284444	0.0166667
	3	1	2	1	2	20	4500	0.0282222	0.0166667
Female	0	0	4	0	8	16	4500	0.0411111	0.0125000
	1	0	3	0	6	18	4500	0.0131111	0.0125000
	2	0	2	2	4	20	4500	0.0051111	0.0125000
	3	1	2	1	2	20	4500	0.0928889	0.0166667
Non-Hispanic White	0	0	4	4	8	16	4500	0.0002222	0.0125000
	1	1	4	1	6	16	4500	0.2251111	0.0166667
	2	1	3	1	4	18	4500	0.3786667	0.0166667
	3	1	2	1	2	20	4500	0.7557778	0.0166667
Non-Hispanic Black	0	0	4	0	8	16	4500	0.0231111	0.0125000
	1	0	3	0	6	18	4500	0.0533333	0.0125000
	2	0	2	0	4	20	4500	0.4664444	0.0125000
	3	0	1	0	2	22	4500	0.7244444	0.0125000
Hispanic or Latino	0	0	4	0	8	13	4500	0.4415556	0.0125000
	1	0	3	0	6	15	4500	0.3024444	0.0125000
	2	0	2	0	4	17	4500	0.4428889	0.0125000
	3	0	1	0	2	19	4500	0.4115556	0.0125000
Northeast	0	0	4	0	8	16	4500	0.1706667	0.0125000
	1	0	3	0	6	18	4500	0.1451111	0.0125000
	2	0	2	0	4	20	4500	0.0586667	0.0125000
	3	0	1	0	2	22	4500	0.0197778	0.0125000
Midwest	0	0	4	0	8	16	4500	0.0700000	0.0125000
	1	0	3	0	6	18	4500	0.0351111	0.0125000
	2	0	2	0	4	20	4500	0.0180000	0.0125000
	3	0	1	0	2	22	4500	0.0197778	0.0125000
South	0	0	4	4	8	16	4500	0.0028889	0.0125000
	1	1	4	1	6	16	4500	0.2871111	0.0166667
	2	1	3	1	4	18	4500	0.4182222	0.0166667
	3	1	2	1	2	20	4500	0.3044444	0.0166667
West	0	0	4	4	8	16	4500	0.0037778	0.0125000
	1	1	4	1	6	16	4500	0.2126667	0.0166667
	2	1	3	1	4	18	4500	0.0995556	0.0166667
	3	1	2	1	2	20	4500	0.0264444	0.0166667

Appendix D

**Table 7:** Annual hemochromatosis-related deaths (ICD-10 E83.1), United States, 1999-2024, by demographic, geographic, age-group, urbanization, and comorbidity stratum. Counts extracted from CDC WONDER Multiple Cause of Death exports. Counts extracted from CDC WONDER Multiple Cause of Death exports (Bridged-Race file 1999–2020; Single-Race file 2021–2024) [22]. Annual cells with fewer than 10 deaths are suppressed in CDC WONDER exports and appear as em-dash (—) per NCHS confidentiality rules. Totals for comorbidity columns use CDC WONDER's "Total" row, which is computed from unsuppressed data and may exceed the sum of visible annual values; totals for other strata are sums of visible annual values. Differences from the overall national total of 16,113 deaths are documented in the workbook Readme. Abbreviations: NH, non-Hispanic; Lg, large; Cent, central; Urb, urbanization; Comorb, comorbidity

Year	Overall	Male	Female	NH White	NH Black	Hispanic	Northeast	Midwest	South	West	Age 15-24	Age 25-34	Age 35-44	Age 45-54	Age 55-64	Age 65-74	Age 75-84	Age 85+	Urb: Lg Cent Metro	Urb: Lg Fringe Metro	Urb: Medium Metro	Urb: Small Metro	Urb: Micropolitan	Urb: NonCore	Comorb: Chronic liver disease	Comorb: Diabetes mellitus	Comorb: Liver cancer (hepatocell	Comorb: Heart failure	Comorb: Sepsis	Comorb: Viral hepatitis C	Comorb: Cardiomyopathy	Comorb: Gout
1999	562	346	206	489	44	16	119	126	183	134	10	15	39	62	87	163	131	40	146	128	121	55	66	46	258	93	61	61	47	23	38	0
2000	550	345	193	465	52	20	111	128	175	136	—	18	29	64	95	144	143	40	138	105	130	70	65	42	253	78	57	64	33	22	33	0
2001	570	357	201	480	40	22	100	145	176	149	—	15	24	59	87	156	149	57	127	112	131	71	68	61	260	75	67	57	41	16	38	—
2002	569	395	165	497	41	21	112	129	197	131	—	12	19	78	91	148	165	47	156	129	127	63	54	40	255	97	68	69	42	21	31	0
2003	531	349	164	450	35	24	112	105	181	133	—	15	25	60	107	130	130	46	130	118	128	51	60	44	230	76	62	59	36	19	32	0
2004	539	353	176	466	35	27	111	120	187	121	—	10	32	76	90	124	140	51	116	111	117	76	56	63	251	92	58	59	30	17	25	—
2005	617	400	203	537	48	16	111	136	220	150	10	—	36	73	126	151	151	51	130	136	147	77	78	49	279	92	69	77	43	26	37	—
2006	573	374	183	496	38	23	105	130	195	143	—	—	33	64	110	118	174	50	138	123	128	66	75	43	262	103	78	71	40	22	23	—
2007	523	355	162	445	33	28	90	116	179	138	10	—	18	86	99	115	131	47	126	114	117	54	58	54	248	84	61	60	41	39	26	—
2008	577	374	196	496	42	21	96	145	186	150	—	13	23	79	102	149	148	50	130	121	124	72	79	51	278	92	48	73	41	36	31	—
2009	560	380	160	473	39	26	113	119	175	153	—	10	20	81	109	146	133	44	134	127	144	52	57	46	264	88	53	44	47	35	27	—
2010	566	372	189	471	50	28	99	130	204	133	—	—	27	77	119	116	140	56	124	119	142	67	73	41	265	82	69	65	39	36	30	—
2011	549	378	159	468	46	22	121	110	163	155	—	13	29	73	130	114	119	55	129	127	128	60	65	40	243	96	64	54	43	32	22	—
2012	542	344	181	454	46	25	87	124	187	144	—	10	27	71	125	116	101	66	116	132	137	57	57	43	259	79	53	46	44	32	16	—
2013	574	370	189	479	46	23	101	134	179	160	—	18	29	60	121	147	101	76	130	128	117	80	69	50	290	79	63	53	38	41	20	—
2014	587	399	184	508	37	26	124	131	185	147	—	14	30	61	151	147	105	61	121	134	141	74	69	48	289	61	65	45	37	43	18	0
2015	565	373	181	501	34	18	102	113	211	139	—	11	18	60	119	154	127	60	120	114	148	74	48	61	268	79	46	52	41	25	26	—
2016	620	407	198	536	39	28	93	140	221	166	—	11	28	58	141	173	137	58	126	142	155	73	65	59	328	77	85	62	57	44	19	—
2017	612	417	184	515	41	32	112	130	206	164	0	17	23	64	125	191	119	57	146	144	143	56	77	46	296	74	70	46	53	30	21	—
2018	654	429	214	561	38	30	98	146	233	177	—	11	20	46	170	166	156	70	141	140	156	75	98	44	310	87	61	71	56	35	27	—
2019	665	450	201	572	51	27	130	156	214	165	—	13	28	53	136	195	152	73	126	139	171	77	85	67	307	96	70	83	44	29	22	—
2020	745	487	244	646	46	39	154	152	240	199	—	15	31	70	155	206	173	88	126	188	197	92	85	57	314	124	88	67	71	34	22	—
2021	820	539	281	730	46	24	132	177	278	233	0	20	43	79	150	223	212	90	152	184	216	94	107	67	352	135	73	88	65	21	20	—
2022	796	552	244	685	45	31	142	156	286	212	—	13	30	63	175	206	209	97	124	207	210	91	106	58	349	129	87	90	44	28	31	—
2023	806	563	243	721	33	31	134	156	281	235	—	12	30	57	141	234	205	117	149	203	194	105	99	56	342	105	81	93	63	21	21	—
2024	841	557	284	742	41	33	155	169	317	200	—	14	25	51	165	244	219	113	162	194	193	96	110	86	362	135	82	106	57	18	18	—
Total	16113	10665	5185	13883	1086	661	2964	3523	5459	4167	30	300	716	1725	3226	4176	3870	1660	3463	3619	3862	1878	1929	1362	7412	2408	1739	1715	1193	745	674	50

Appendix E 

**Table 8:** Hemochromatosis-related deaths (ICD-10 E83.1) and age-adjusted mortality rates by U.S. state, 1999–2020 and 2021–2024, and combined totals. Age-adjusted mortality rates are per 100,000 population, standardized to the 2000 U.S. standard population using the direct method [23]. AAMRs shown are period-specific as reported by CDC WONDER; a single combined 1999–2024 AAMR cannot be computed from these two sub-period files without age-stratified data, so combined deaths and the percentage of the national total are reported instead. Four jurisdictions (North Dakota, South Dakota, Delaware, District of Columbia) had fewer than 10 deaths during 2021-2024 and were suppressed in that period's export per NCHS confidentiality rules; those states' 2021–2024 deaths appear as em-dash. Combined state totals sum to 16,084 (99.8% of the 16,113 national total); the 29-death shortfall reflects the four suppressed jurisdictions. States are ranked by combined total deaths. Abbreviations: AAMR, age-adjusted mortality rate; CI, confidence interval

State	Deaths 1999–2020	AAMR 1999–2020 (95% CI)	Deaths 2021–2024	AAMR 2021–2024 (95% CI)	Total deaths 1999–2024	% of U.S. total	Rank (by total deaths)
California	1,358	0.15 (0.14–0.16)	244	0.14 (0.12–0.16)	1,602	9.9	1
Florida	786	0.14 (0.13–0.16)	250	0.20 (0.17–0.22)	1,036	6.4	2
Texas	803	0.15 (0.14–0.16)	201	0.17 (0.15–0.19)	1,004	6.2	3
New York	637	0.11 (0.10–0.12)	148	0.13 (0.11–0.16)	785	4.9	4
Pennsylvania	605	0.17 (0.15–0.18)	162	0.22 (0.19–0.26)	767	4.8	5
Ohio	563	0.21 (0.19–0.23)	104	0.17 (0.14–0.21)	667	4.1	6
North Carolina	432	0.19 (0.17–0.21)	101	0.21 (0.17–0.25)	533	3.3	7
Washington	411	0.26 (0.23–0.28)	122	0.31 (0.26–0.38)	533	3.3	8
Michigan	422	0.17 (0.15–0.19)	87	0.16 (0.12–0.20)	509	3.2	9
Massachusetts	353	0.21 (0.19–0.24)	94	0.26 (0.21–0.32)	447	2.8	10
Tennessee	348	0.22 (0.19–0.24)	96	0.25 (0.20–0.31)	444	2.8	11
Illinois	369	0.11 (0.10–0.12)	64	0.09 (0.06–0.11)	433	2.7	12
Oregon	323	0.34 (0.30–0.38)	107	0.47 (0.38–0.57)	430	2.7	13
Colorado	278	0.26 (0.22–0.29)	128	0.48 (0.40–0.57)	406	2.5	14
Virginia	315	0.18 (0.16–0.20)	85	0.19 (0.15–0.24)	400	2.5	15
Minnesota	295	0.22 (0.20–0.25)	87	0.30 (0.24–0.37)	382	2.4	16
Arizona	268	0.17 (0.15–0.19)	87	0.20 (0.15–0.25)	355	2.2	17
New Jersey	289	0.11 (0.10–0.13)	58	0.12 (0.09–0.16)	347	2.2	18
Missouri	258	0.17 (0.15–0.19)	73	0.23 (0.18–0.30)	331	2.1	19
Maryland	231	0.17 (0.14–0.19)	82	0.26 (0.20–0.33)	313	1.9	20
Georgia	240	0.10 (0.08–0.11)	54	0.11 (0.08–0.14)	294	1.8	21
Wisconsin	218	0.15 (0.13–0.17)	75	0.23 (0.18–0.29)	293	1.8	22
Indiana	211	0.14 (0.12–0.16)	53	0.15 (0.11–0.21)	264	1.6	23
South Carolina	199	0.16 (0.14–0.19)	56	0.18 (0.13–0.25)	255	1.6	24
Iowa	195	0.24 (0.21–0.28)	44	0.27 (0.19–0.37)	239	1.5	25
Oklahoma	181	0.20 (0.17–0.23)	54	0.28 (0.21–0.37)	235	1.5	26
Kentucky	180	0.16 (0.14–0.18)	47	0.21 (0.15–0.28)	227	1.4	27
Alabama	183	0.16 (0.13–0.18)	37	0.15 (0.10–0.21)	220	1.4	28
Connecticut	152	0.16 (0.13–0.19)	28	0.16 (0.11–0.23)	180	1.1	29
Kansas	124	0.17 (0.14–0.20)	36	0.24 (0.16–0.34)	160	1.0	30
Nevada	117	0.20 (0.16–0.23)	37	0.25 (0.18–0.35)	154	1.0	31
Arkansas	114	0.15 (0.12–0.18)	27	0.17 (0.11–0.26)	141	0.9	32
New Mexico	114	0.24 (0.19–0.29)	27	0.24 (0.16–0.36)	141	0.9	33
Idaho	96	0.28 (0.22–0.34)	41	0.42 (0.30–0.58)	137	0.9	34
West Virginia	101	0.19 (0.15–0.23)	31	0.31 (0.21–0.46)	132	0.8	35
Maine	105	0.28 (0.22–0.33)	23	0.24 (0.15–0.41)	128	0.8	36
Rhode Island	112	0.41 (0.34–0.49)	12	0.19 (0.10–0.38)	124	0.8	37
Nebraska	104	0.24 (0.20–0.29)	18	0.19 (0.11–0.31)	122	0.8	38
Utah	95	0.20 (0.16–0.25)	25	0.19 (0.12–0.29)	120	0.7	39
New Hampshire	89	0.27 (0.21–0.33)	23	0.27 (0.17–0.44)	112	0.7	40
Louisiana	86	0.09 (0.07–0.12)	19	0.08 (0.04–0.13)	105	0.7	41
Montana	79	0.30 (0.24–0.38)	21	0.33 (0.20–0.54)	100	0.6	42
Hawaii	64	0.18 (0.14–0.24)	12	0.16 (0.08–0.30)	76	0.5	43
Wyoming	57	0.43 (0.32–0.56)	19	0.66 (0.39–1.09)	76	0.5	44
Vermont	59	0.34 (0.26–0.44)	15	0.43 (0.23–0.79)	74	0.5	45
Mississippi	56	0.07 (0.05–0.09)	10	0.08 (0.04–0.15)	66	0.4	46
South Dakota	57	0.24 (0.18–0.32)	—	—	57	0.4	47
North Dakota	49	0.28 (0.20–0.37)	—	—	49	0.3	48
Alaska	27	0.22 (0.14–0.34)	10	0.33 (0.15–0.65)	37	0.2	49
Delaware	32	0.13 (0.09–0.19)	—	—	32	0.2	50
District of Columbia	10	—	—	—	10	0.1	51
